# Metabolic–immune crosstalk in osteosarcoma: mechanisms and therapeutic opportunities

**DOI:** 10.3389/fimmu.2026.1818745

**Published:** 2026-05-29

**Authors:** Xihan Wan, Wenge He, Baicheng He, Liang Chen

**Affiliations:** 1 Key Laboratory of Biochemistry and Molecular Pharmacology of Chongqing, Chongqing Medical University, Chongqing, China; 2 Department of Bone and Soft Tissue Tumors, Chongqing University Cancer Hospital, School of Medicine, Chongqing University, Chongqing, China; 3 Chongqing Key Laboratory of Translational Research for Cancer Metastasis and Individualized Treatment, Chongqing University Cancer Hospital, Chongqing, China; 4 Department of Pharmacology, School of Pharmacy, Chongqing Medical University, Chongqing, China

**Keywords:** combination therapy, immunotherapy, metabolic reprogramming, osteosarcoma, tumor immune microenvironment

## Abstract

Osteosarcoma (OS) is a highly aggressive primary bone malignancy that predominantly affects children and adolescents. Although surgical intervention and neoadjuvant therapy demonstrate efficacy in localized OS, postoperative survival rates remain suboptimal for patients with metastatic and recurrent OS. In recent years, immunotherapy has garnered considerable attention due to its promising efficacy across various solid tumors. However, the distinct immunosuppressive tumor microenvironment in OS restricts therapeutic response, as immunosuppressive states are further intensified in metastatic and recurrent lesions, thereby complicating immunotherapy efforts. Recent studies have found that metabolic reprogramming plays a crucial role in shaping the immunosuppressive tumor microenvironment of OS. Tumor cells can induce adaptive metabolic changes in immune cells through competitively consuming nutrients, accumulating immunosuppressive metabolites, and secreting exosomes. This subsequently diminishes their immune functions and promotes immune escape, which partially explains the poor efficacy of immunotherapy in OS. Thus, combining immunotherapy and metabolic-targeted drugs is a potential strategy for enhancing treatment efficacy against OS. This review focuses on the major characteristics of the immunosuppressive microenvironment in OS. It details how metabolic reprogramming in glucose, lipid, and amino acid metabolism remodels this environment and influences key immune cells, including tumor-associated macrophages (TAMs), T cells, and natural killer (NK) cells. It further explores the translational potential of combining metabolic interventions with immunotherapy to advance the clinical application in OS.

## 1 Introduction

Osteosarcoma (OS) is the most prevalent primary malignant bone tumor, originating from mesenchymal stem cells (1). Its incidence rate is only 3–5 cases per million annually, yet it is associated with high rates of disability and mortality (2). OS predominantly affects children and adolescents, with approximately 60% of cases occurring in individuals aged 10 to 20 years, and stands as the second leading cause of cancer-related death in this demographic (3). Although OS can arise in any bone, it shows a strong predilection for the metaphyses of long bones in the extremities, most commonly the distal femur and proximal tibia (4).

The standard treatment regimen for OS remains neoadjuvant chemotherapy-surgery-consolidation chemotherapy. This comprehensive, long-term treatment approach can improve the 5-year survival rate of localized OS patients to approximately 60% (5). Currently, high-dose Methotrexate, Doxorubicin, and Cisplatin are routinely administered as the standard treatment in most clinical settings, known as the neoadjuvant MAP regimen. However, this regimen still faces numerous challenges. A prospective randomized trial based on the MAP regimen clearly demonstrated that postoperative modification and intensification of therapy could not further improve long-term outcomes for patients with poor responses (6). Meanwhile, in clinical practice, OS carries a high risk of early hematogenous metastasis and recurrence, with a recurrence and metastasis rate of 20% (5). For OS patients with extensive metastasis and recurrence, prognosis remains very poor even after MAP chemotherapy and local tumor control, with long-term survival rates <30% (7). Current evidence indicates that chemotherapy-centered approaches have reached an efficacy plateau for metastatic and recurrent OS, underscoring an urgent need for novel therapeutic strategies.

Current research focuses on immunotherapy, which has demonstrated promising efficacy in other solid tumors. Over the past decade, immunotherapy represented by immune checkpoint inhibitors (ICIs) and adoptive cell therapy has achieved breakthrough advances in various tumors. The clinical success of immune ICIs is evident in malignancies such as melanoma, non-small cell lung cancer, and renal cell carcinoma (8). Similarly, chimeric antigen receptor T (CAR-T) cells have shown remarkable efficacy in treating hematological cancers, including acute lymphoblastic leukemia and non-Hodgkin lymphoma (9). However, clinical trials exploring immunotherapy in OS have demonstrated limited progress. Current clinical evidence suggests that PD-1/PD-L1 inhibitors have yet to demonstrate significant improvement in objective response rate, progression-free survival, or overall survival for OS patients (10, 11). With the application of single-cell omics technology, single-cell sequencing analysis of OS has revealed significant infiltration of immunosuppressive cells within its immune microenvironment, predominantly characterized by exhausted CD8+ T cells and M2-type tumor-associated macrophages (TAMs), which exhibit high expression of immunosuppressive receptors such as TIGIT and PD-1. However, the infiltration of M1-type TAMs and activated T cells in the tumor immune microenvironment(TIME) of OS is insufficient (2). These results suggest that the poor clinical efficacy of immunotherapies such as ICI in OS may be associated with the ‘cold’ tumor immune microenvironment caused by the low immunogenicity of OS and reduced infiltration of activated T cells.

Metabolic reprogramming refers to the adaptive transformation of cellular metabolic pathways under specific physiological and pathological conditions to meet their own growth and functional needs. It is now regarded as a key mechanism supporting immune cell dysfunction. Specifically, it drives the depletion, functional inhibition and acquisition of immunosuppressive phenotypic transformation of various immune cells (12). Tumor cells often undergo metabolic reprogramming to adapt to the fluctuating tumor immune microenvironment, thereby regulating growth, invasion, and migration. This adaptation also constitutes a significant cause of resistance to anticancer drugs (13). The immunological activity of OS is tightly linked to the metabolic reprogramming of immune cells and OS cells in the TIME (14). This also suggests that improving metabolic reprogramming in OS metastases is a potential strategy to enhance the efficacy of immunotherapy.

Accordingly, this article provides a comprehensive overview of the immune landscape within the OS tumor microenvironment and its prognostic significance. Building on this foundation, the review will focus on the key features of metabolic reprogramming in OS involving glucose, lipids, and amino acids. We will examine how these alterations dynamically regulate the function of TAMs, T cells, and NK cells through mechanisms like nutrient competition, metabolite accumulation, and exosomal signaling. Finally, we will discuss the potential advantages and challenges of combining targeted metabolic reprogramming with immunotherapeutic strategies such as ICI and CAR-T cell therapy, aiming to provide new theoretical foundations and translational perspectives for enhancing the clinical efficacy of OS immunotherapy.

## 2 Overview of tumor immune microenvironment

The TIME is a complex ecosystem characterized by immunosuppression, comprising immune cells, stromal cells, and acellular components (15). Cellular components within the OS immune microenvironment include immune cells such as TAMs, tumor-associated neutrophils (TANs), dendritic cells (DCs), T cells, B cells, and NK cells. Some single-cell RNA sequencing studies on OS have revealed that the TIME of OS is predominantly composed of myeloid cells rather than lymphocytes. In patients with aggressive, immune checkpoint inhibitor-resistant OS exhibiting poor prognosis, significantly reduced infiltration was observed in immature B cells, activated B cells, central memory CD4+ T cells, CD8+ T cells, M1-type TAMs, myeloid-derived suppressor cells (MDSCs), NK cells, NKT cells and neutrophils (16). Among these immune cells, the levels and characteristics of macrophages and T cells are closely related to key events of poor prognosis in OS such as metastasis and chemotherapy resistance. These findings indicate that the degree and composition of immune cell infiltration significantly impact the prognosis and clinical behavior of OS (17).

Different types of OS also exhibit heterogeneity in immune cell infiltration. Compared to primary OS, pulmonary metastases demonstrate higher density of M2-type TAMs, while the proportion of CD4+ and CD8+ T cells is lower than that in primary lesions (2, 18). The heterogeneity of the OS immune microenvironment is closely related to the complex and diverse functions of immune cells. In recent years, many studies have analyzed the characteristics of immune cell infiltration in the OS microenvironment (**Table 1**).

**Table 1:** Features of immune cell infiltration in the TIME of OS.

Species	Analytical subjects	Analytical methods	Characteristics of immune infiltration	Ref
Human	6 OS biopsy sample and 6 OS samples from GEO database(GSE162454)	scRNA-seq + Spatial transcriptomics	Primary tumor: ↑: MDSC, Treg, exhausted CD8+ T cell, LAMP3+ DC	(19)
Human	82 OS samples from the TARGET database and 37 OS samples from the GEO database (GSE39055)	Bulk RNA-seq + Immune infiltration estimation (CIBERSORT) +Survival analysis(Univariate Cox regression analyses)	Primary tumor: ↑: TAMs, CD8+ T cells, NK cells, Th2 ↓: Mast cells and Tregs Poor prognosis: ↓: TAMs, T cells, Th1 cells, DCs, TANs, TFH cells, iDCs, NK cells and Tregs ↑: Th2 cells, γδ T cells	(20)
Human	119 OS samples from the TCGA database	Bulk RNA-seq + Immune infiltration estimation (CIBERSORT/ssGSEA/MCP-counter, etc.)	Male patients : ↑: Activated B cells, CD8+ T cells, Monocytes, NK cells	(21)
Human	85 OS samples from the TARGET database and 53 OS samples from the GEO database (GSE21257)	Bulk RNA-seq + Immune infiltration estimation (CIBERSORT) + risk model	Poor prognosis: ↑:iDCs	(22)
Human	88 OS samples from the TCGA database	Bulk RNA-seq + Metagene Approach and Deconvolution Method(TIMER 2.0)	Metastatic tissues: ↑: M2-type TAMs ↓: CD56^bright^NK cells, immature B cells, M1-type TAMs and TANs	(23)
Human	11 advanced OS samples (7 primary, 2 recurrent, 2 pulmonary metastatic)	scRNA-seq + DEG analysis + Clustering (t-SNE/UMAP) + GSEA +GSVA	Primary tumor: ↑:Exhausted T cells Metastatic tissue: ↑: pro-inflammatory FABP4+ TAMs, M2-type TAMs ↓: T cells	(2)
Human	84 OS samples from the TARGET database and 140 OS samples from the GEO database (GSE21257, GSE16091, GSE33382)	Bulk RNA-seq + ssGSEA	High TMEindex group: ↓: Activated B cells, immature B cells, central memory CD4+ and CD8+T cells,TAM, MDSC, NK cells, NKT cells, Monocytes, neutrophils, Tregs, Th1	(16)

Thus, in-depth insights into immune cell mechanisms constitute a critical foundation for both comprehending the heterogeneity of OS and rationally devising immunotherapies that target specific cellular players within the TIME.

### 2.1 TAMs

As important innate immune cells, macrophages are generally considered to be differentiated from hematopoietic stem cells and circulating monocytes. During inflammatory and immune responses, they are recruited to relevant sites to phagocytose and clear pathogens, necrotic cells, and tissue debris (24).

TAMs are the predominant immune cell type in the OS microenvironment, representing up to 50% of the total immune infiltrate (25). TAMs exhibit significant functional heterogeneity in the TIME of OS. This is primarily manifested by a spectrum of phenotypes, ranging from pro-tumoral M2 phenotype and anti-tumoral M1 phenotype to transitional states expressing markers of both (26). M1-type TAMs possess tumor-suppressive functions. They can be activated by interferon-γ (IFN-γ) or lipopolysaccharide (LPS), and are characterized by high expression of MHC class II molecules, along with strong phagocytic and tumor-killing capabilities. Conversely, M2-type tumor-associated macrophages tend to promote tumor progression. They form under stimulation by interleukin-4 (IL-4) and similar factors, manifesting as lower MHC class II expression levels and the ability to promote angiogenesis and metastasis (27).

M1-type and M2-type TAMs in OS display contrasting metabolic and functional profiles. In contrast to glycolysis-dependent M1-type TAMs, M2-type TAMs rely more on OXPHOS and fatty acid oxidation(FAO) (28). Recent research has demonstrated that increased infiltration of M2-type TAMs and elevated expression of their marker CD163 in the TIME of OS are associated with poor patient prognosis (29). Further findings revealed that CD163+ M2-type TAMs can significantly suppress T cell function. Thus, the depletion of M2-type TAMs leads to a more favorable immune landscape, resulting in enhanced T cell infiltration and a rise in pro-inflammatory cytokine secretion (30).

Several single-cell sequencing studies based on osteosarcoma patients also indicate that TAMs play a crucial role in metastatic OS. Compared to primary OS, pulmonary metastatic lesions exhibit higher density of M2-type TAMs and demonstrate greater invasive capacity (18). Studies demonstrate that TAMs within pulmonary metastases highly express PD-1. Anti-PD-1 antibody therapy increases the infiltration of anti-tumor M1-type TAMs while reducing pro-tumor M2-type TAMs, enhances NK cell infiltration, and consequently significantly inhibits pulmonary metastasis in OS (31). Furthermore, the infiltration of CCL18+ TAMs is markedly elevated in metastatic OS tissues compared with their primary counterparts. Through abundant secretion of CCL18, TAMs can further promote the metastasis and growth of OS (32). The phenotypic and functional differences in TAMs between primary lesions and metastatic lesions may be associated with the characteristics of the tumor immune microenvironment at distinct anatomical sites.

### 2.2 TANs

Neutrophils are the most abundant leukocytes in human peripheral blood, representing 60-70% of total peripheral blood leukocytes in adults, and serve as key mediators of anti-infection immunity and inflammatory responses (33). In recent years, neutrophils have garnered increasing attention in cancer research. Neutrophils within the tumor immune microenvironment are termed TANs, which exhibit phenotypic heterogeneity and functional diversity. Based on their roles in TIME, TANs can be classified into anti-tumor (N1) and pro-tumor (N2) phenotypes (34). In OS, the N1 subtype of TANs may be more prevalent in early disease stages and is associated with favorable treatment responses and prognosis; conversely, the N2 subtype may foster an immunosuppressive tumor microenvironment that suppresses the efficacy of immunotherapy (35). Yang et al. found that in patients with OS, the metastatic tissues of OS exhibit higher TANs infiltration ratios compared to non-metastatic tumor tissues. Concurrent analyses using TIMER and GEPIA revealed that genes associated with favorable prognosis (e.g., LPAR5, EVI2B) show positive correlation with TANs infiltration (23). However, this study did not analyze the subtype infiltration of TANs in different tumor microenvironments, which may be related to the difficulty in identifying biomarkers among different subtypes. The mechanism by which TANs promote proliferation and metastasis in OS may involve their interaction with the bone matrix and immunoregulatory functions (36). The presence of a low molecular weight cyclin E1 (LMW-Cyclin E1) isoform in OS is linked to key malignant phenotypes such as enhanced proliferation, therapy resistance, and poor prognosis. TANs can release neutrophil elastase to cleave full-length Cyclin E1, generating LMW-Cyclin E1, thereby enhancing tumor invasion and metastasis capabilities. Depleting TANs can inhibit tumor growth and angiogenesis, representing a potential therapeutic strategy for OS (37).

Components associated with TANs also receive significant attention in tumor research. Neutrophil Extracellular Traps (NETs) are web-like structures released by activated neutrophils, composed of DNA, histones, and other components (38). Mounting evidence suggests that NETs may serve as one mechanism through which TANs promote OS growth, angiogenesis, and pulmonary metastasis. However, the precise mechanisms underlying NET-mediated promotion of malignant behaviors in OS remain incompletely understood. NET-based prognostic prediction models for OS are gradually emerging. High expression of NET-related genes correlates with reduced immune infiltration and down-regulated immune checkpoint expression, wherein CFH and ATG7 are regarded as potential immune checkpoint-like molecules or autophagy-related therapeutic targets (39–41). This suggests that NETs may regulate anti-tumor immune responses by affecting the functions of other immune cells such as T cells, NK cells, and TAMs.

### 2.3 DCs

As highly efficient antigen-presenting cells originating from hematopoietic stem cells, DCs play a critical role in initiating immune responses (42). Based on function and surface markers, DCs are broadly divided into three major subsets, including plasmacytoid DCs (pDCs), conventional DC1 (cDC1), and conventional DC2 (cDC2) (43).

Conventional DCs in primary OS patients primarily comprise two subsets: cDC1 and cDC2 (44). Functionally, the cDC1 subset plays a critical role in T cells activation and enhancement of anti-tumor immunity (2). The cDC2 subset is closely associated with immune modulation and inflammatory response, the precise mechanisms of which remain under active investigation. High levels of infiltration of immature DCs generally indicate reduced immune reactivity in OS patients and are associated with poor prognosis (22). Furthermore, high-risk OS patients exhibit elevated pDC levels alongside reduced proportions of cDCs and their immature subtypes within the TIME, a phenotype correlated with poor prognosis (20). Advanced analysis of DC subsets based on molecular markers (CD14/CD163, cDC1, cDC2, CCR7) revealed increased cDC2 abundance in metastatic pulmonary lesions versus primary and recurrent OS, suggesting a dynamic shift in DC subset distribution during disease progression and metastasis (2).

DC vaccines effectively inhibit pulmonary metastasis of OS in mice. Combination with agents such as anti-transforming growth factor-beta (TGF-β)/glucocorticoid-induced TNF receptor (GITR) antibodies can further enhance anti-tumor immunity (45, 46). However, the clinical efficacy of DC vaccines in treating OS remains suboptimal, which is likely due to T cell exhaustion in patients and the relatively weak targeting specificity of the vaccines themselves. Combination with other immunotherapies or enhancing the targeting specificity of DC vaccines may represent future therapeutic strategies.

### 2.4 MDSCs

MDSCs are defined as a heterogeneous group of immature myeloid cells of hematopoietic origin, characterized by their potent immunosuppressive capacity. They can further differentiate into TAMs, TANs, and DCs (47). Defined by Ly6G and Ly6C expression, MDSCs are categorized into polymorphonuclear (PMN-MDSC) and monocytic (M-MDSC) subsets. PMN-MDSCs are the predominant population, representing over 75% of all MDSCs, compared to the 10-20% represented by M-MDSCs (48). Single-cell RNA sequencing analysis in OS patients has revealed that MDSCs represent a major cellular component contributing to the formation of the immunosuppressive tumor microenvironment (19).

MDSCs promote tumor progression and metastasis by interacting with various immune cells within the OS tumor immune microenvironment, facilitating NF-κB upregulation and the formation of an immunosuppressive microenvironment (19). Furthermore, MDSC-mediated regulation of T cells is particularly crucial. MDSCs are recruited and survive in OS through the SDF-1/CXCR4 axis, inhibiting the proliferation of CD8+ T cells, and are a key factor leading to resistance to anti-PD-1 treatment (49). Concurrently, MDSCs release molecules like VEGF and TGF-β to promote tumor angiogenesis and pre-metastatic niche formation, potentially facilitating pulmonary metastasis in OS (50).

### 2.5 T cells

As key mediators of both cellular and humoral immunity, T lymphocytes are broadly classified by function into CD4+ helper T cells, CD8+ cytotoxic T cells (CTLs), and regulatory T cells (Tregs) (51). Playing a central role in tumor immunology, T cells are a key research focus. In the TIME, they commonly develop a specialized dysfunctional state termed ‘exhaustion,’ which is characterized by the gradual decline of effector functions in CD8+ T cells under persistent antigenic stimulation (52). Characteristics of T cell exhaustion encompass upregulation of multiple inhibitory receptors (e.g., PD-1, LAG-3, TIM-3), diminished effector functions following TCR signaling stimulation, and reduced proliferative capacity. These alterations ultimately culminate in impaired anti-tumor immune responses, fostering an immunosuppressive TIME (53). Furthermore, exhausted T cells exhibit metabolic dysfunction characterized by suppressed mitochondrial respiration and glycolytic processes, which may contribute to their suboptimal response to immunotherapy (54).

Research indicates that within the TIME of OS, T cells represent one of the predominant infiltrating immune cells, second only to macrophages, playing a pivotal role in anti-tumor immunity. However, further analysis revealed that the primary infiltrating cells in primary OS tissues are predominantly exhausted CD8+ T cells (2). The quantities of TIM-3+PD-1- T cells and TIM-3+PD-1+ T cells in OS tissues are also significantly higher than those in peripheral blood (30). OS cells can suppress T cell function by overexpressing immunosuppressive molecules such as PD-L1, thereby achieving immune escape (55). Simultaneously, other immunosuppressive cells within the TIME of OS such as M2-type TAMs and MDSCs further suppress T cell function, forming a vicious cycle (56).

The quantity and proportion of T cells within the TIME may also influence the metastasis and recurrence processes of OS. While studies show a lower proportion of CD4+ and CD8+ T cells in metastatic/recurrent OS lesions compared to primary tumors, the absolute T cell count is significantly higher in metastatic sites relative to both primary and locally recurrent lesions (2, 57).

Given the functional exhaustion and insufficient infiltration of T cells within the OS immune microenvironment, adoptive T cell immunotherapy holds considerable potential. However, the hypoxic and acidic microenvironment formed by high-density OS cells and immunosuppressive cells such as M2-type TAMs and MDSCs can severely impair the efficacy of CAR-T cell immune responses, constituting a major obstacle to the application of T cell immunotherapy in OS (58).

### 2.6 NK cells

NK cells are innate lymphocytes derived from hematopoietic stem cells. They are distributed in the blood, peripheral lymphoid organs, liver, and spleen, comprising approximately 5-15% of circulating lymphocytes (59). NK cells are recognized as innate cytotoxic lymphocytes capable of rapidly eliminating pathogenic microorganisms or tumor cells without requiring MHC class I molecule involvement or prior antigen sensitization (60). Human NK cells comprise two main subsets with distinct functions and phenotypes, defined by their level of CD56 expression: CD56^bright^ and CD56^dim^ NK cells. The CD56^bright^ subset represents immature NK cells primarily distributed in lymphoid tissues. These cells secrete cytokines such as IFN-γ and tumor necrosis factor-α (TNF-α), promoting tumor cell apoptosis and inhibiting proliferation, though exhibiting limited cytotoxic activity. The CD56^dim^ subset represents mature NK cells, primarily found in peripheral blood, which directly kill tumor cells by releasing perforin, granzymes, TNF-α, and expressing FasL; simultaneously, this subset expresses CD16 receptors that can bind to tumor cells and mediate antibody-dependent cellular cytotoxicity to exert anti-tumor effects (61). NK cells show appreciable enrichment in the TIME of OS. Notably, CD56^dim^ NK cells are more abundant in OS tissues of male patients, highlighting the importance of innate immunity in OS (21). Activated NK cells are significantly enriched in patients with favorable prognosis, whereas patients with poor prognosis exhibit reduced NK cell activity, suggesting an immunosuppressive state. This indicates that activating NK cells may serve as a potential strategy to improve clinical outcomes for patients with highly malignant OS (62). OS cells highly express CD155, which binds to inhibitory receptors TIGIT and CD96 on NK cell membranes while also interacting with the activating receptor DNAM-1 on NK cells. Blocking CD155 promotes degranulation and IFN-γ production in allogeneic NK cells via DNAM-1, enhancing their cytotoxic effects against OS. This approach demonstrates particularly significant efficacy in models of pulmonary metastasis with post-transplantation recurrence (63). Similar to CD155, FSTL1—highly expressed in OS—co-induces NK cell apoptosis and functional suppression through its ligand CD6. Anti-FSTL1 therapy significantly increases activated NK cells within tumors and enhances their anti-tumor effects (64). Additionally, the anti-tumor functionality of NK cells is influenced by PD-1/PD-L1 interactions. Zhang et al. found that after blocking the PD-L1/PD-1 pathway using PD-L1 antibodies, the cytotoxic capability of NK cells to induce specific lysis of human OS cells through secretion of granzyme B was significantly enhanced (65).

## 3 Metabolic reprogramming in the microenvironment of OS

Despite the infiltration of diverse immune cells, the TIME of OS is predominantly composed of immunosuppressive populations, such as M2-type TAMs and MDSCs. In contrast, immune cells with anti-tumor functions, such as CD8+ T cells, M1-type TAMs, and NK cells, exhibit functional suppression and reduced infiltration (66). Additionally, multiple immune cells exhibit high expression of immune checkpoint molecules like PD-L1/PD-1, TIM-3, and TIGIT, suggesting impaired function of immune effector cells (2).

Recent studies have gradually revealed that metabolic reprogramming plays a crucial role in shaping the immunosuppressive microenvironment of OS. To systematically illustrate this intricate interplay, we have summarized key findings from recent multi-omics and prognostic studies (**Table 2**), which highlight the specific metabolic pathways, key molecular mediators, and their associated immune infiltration landscapes in OS. The following will systematically elaborate on how metabolic reprogramming modulates the immunosuppressive microenvironment in OS from three aspects: glucose metabolism, lipid metabolism, and amino acid metabolism.

**Table 2:** Summary of metabolic reprogramming and immune cells in OS.

Species	Metabolic pathway	Key molecules	Immune infiltration and metabolism	Ref
Human	Amino acid metabolism, lipid metabolism, TCA cycle, glucose metabolism	FN1, APOE, CCL3L1, TCF7, FOXP3, etc.	• Naïve T cells Depend on amino acid metabolism to maintain activation potential • NK cells Rely on lipid metabolism and the TCA cycle to support cytotoxic function • C06 macrophages Acquire an immunosuppressive and tissue-repair phenotype through lipid metabolism reprogramming. • C04 Macrophages Exhibit pro-inflammatory characteristics and are associated with pathways such as complement activation.	(12)
Human	Lactic acid metabolism, Glycolysis,	SLC7A7, CYP27A1, etc.	• High-risk group: Characterized by low immune infiltration, predominantly M2-type TAMs. • Low-risk group: Characterized by high immune infiltration, predominantly M1-type TAMs.	(67)
Human	Glycolysis, OXPHOS, Lipid metabolism	PLEK, etc.	• High PLEK expression group: Associated with high infiltration of TAMs, DCs, and CD4^+^ T cells, indicating better prognosis. Single-cell analysis revealed that PLEK is predominantly enriched in macrophage subsets with high metabolic activity (enhanced glycolysis and OXPHOS).	(68)
Human	Glycolysis	SLC16A8, STC2, ABCB6, MXI1, P4HA1, etc.	• High-risk group: Decreased CD8+ T cells, increased naïve CD4+ T cells, resting dendritic cells, and activated mast cells.	(69)
Human	Glycolysis, OXPHOS, FAO		•MPE: Higher proportion of CD8+ T cells in MPE but enhanced glycolysis, attenuated OXPHOS, and functional exhaustion; •PT: Enrichment of M2-type TAMs in PT, metabolic preference for FAO and immunosuppression.	(70)
Human	Hypoxia and lactic acid metabolism.	MAFF, COL5A2, FAM162A, SQOR, etc.	High-risk group: Reduced immune cell infiltration, especially CD8+ T cells, CD4+ T cells, and NK cells;	(71)
Human	Lipid metabolism	ALOX15B, ME1, GPD1, etc.	• High-risk group: Low immune score, high tumor purity, reduced infiltration of B cells, macrophages, dendritic cells, etc. Its lipid metabolism reprogramming characteristics are associated with immune cell dysfunction and insufficient infiltration. • Low-risk group: Higher immune scores, lower tumor purity, extensive immune cell infiltration, and stronger immune activity.	(72)
Human	Macrophage lipid metabolism	ALDH1L2, GAL3ST4, PPARG, etc.	• High-risk group: Weaker immune response and higher tumor purity. Its undifferentiated macrophages (M0-type) are enriched with lipid metabolism genes and pathways. • Low-risk group: High immune infiltration, particularly enriched with polarized macrophages (M1/M2 phenotypes), accompanied by an active immune response. Macrophages with a metabolically differentiated state skewed toward immune activation correlate with favorable chemotherapeutic responses.	(73)
Human	Cuproptosis-Sphingolipid Metabolism Network	B4GALNT1, SGMS2, ABCA2, etc.	• High-risk group: Significantly reduced infiltration of CD8+ T cells and NK cells, coupled with an increase in Tregs. • Low-risk group: Higher CD8+ T cell infiltration correlates with stronger anti-tumor immunity.	(74)
Human	Arachidonic acid metabolism	CD36, CLDN11, STOM, EPYC, PANX3, etc.	• High-risk group: Demonstrated lower immune score, stromal score, and ESTIMATE score; STOM levels correlate positively with NK cells, whereas PANX3 expression is inversely related to central memory CD8+ T cell populations.	(75)
Human	Polyamine metabolism	FAM162A, SIGMAR1, SQLE, etc.	• High-risk group: Reduced immune infiltration with decreased abundance of immune cells such as NK cells, B cells, and T cells; diminished expression of immune checkpoint genes.	(76)

### 3.1 Glucose metabolism

**Figure 1:**
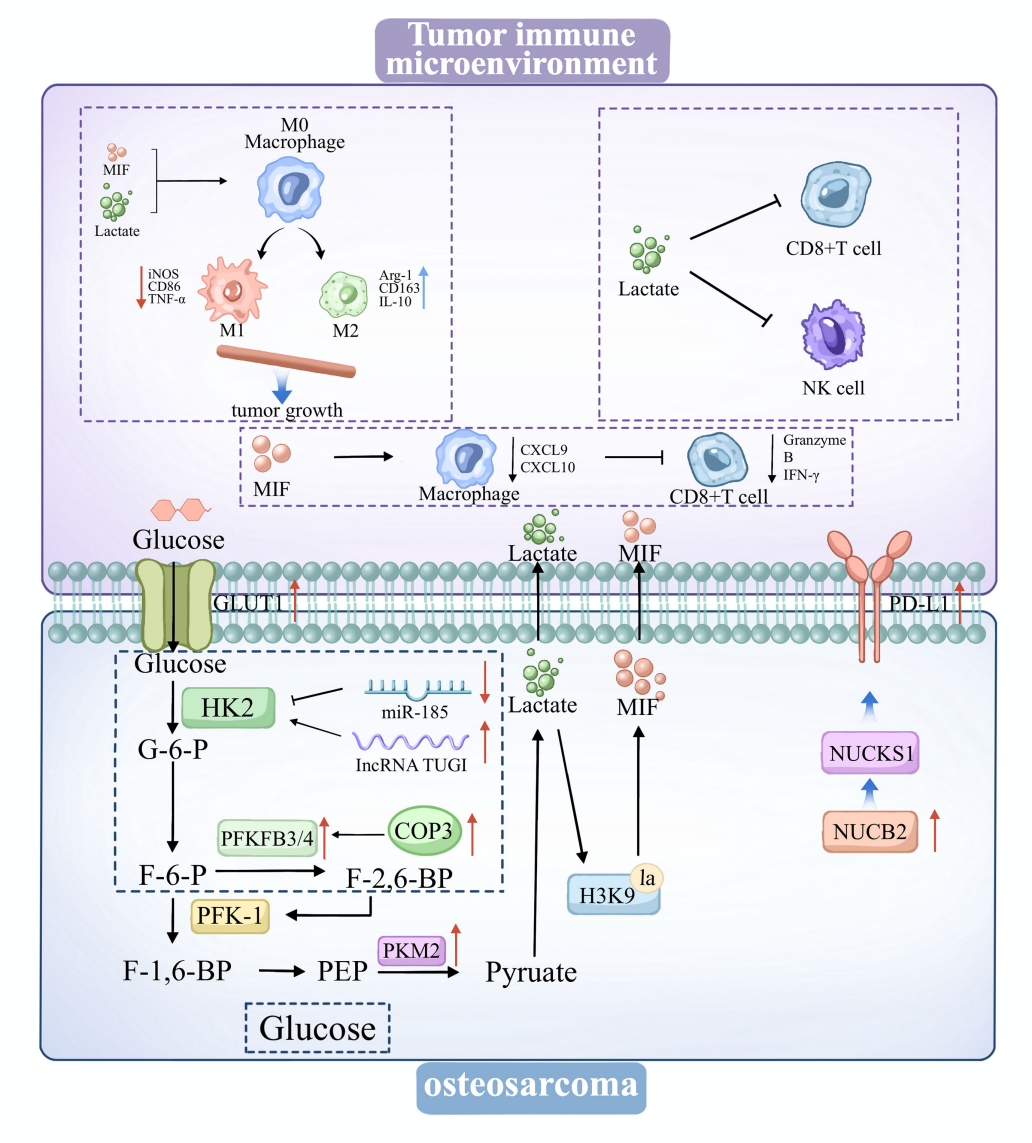
Reprogramming of glucose metabolism in OS and its TIME. Upregulation of glycolytic key enzymes (e.g., HK2, PFKFB3/4, PKM2) in OS cells increases glucose consumption and lactate production. Accumulated lactate within the TIME promotes the polarization of M0 macrophages toward the M2 phenotype while inhibiting the activity of M1-type TAMs, thereby supporting OS growth. Lactate also directly impairs the cytotoxicity of CD8+ T cells and NK cells. Furthermore, lactate accumulation in OS enhances H3K9 lactylation, thereby promoting MIF secretion, which induces the differentiation of TAMs toward the M2 phenotype and reduces the secretion of chemokines such as CXCL9 and CXCL10, subsequently inhibiting CD8+ T cell function. Under glucose-deprived conditions, OS further promotes the expression of PD-L1 on the cell membrane via the NUCB2/NUCKS1 axis, thereby mediating immunosuppression.

In the early 20th century, Otto Warburg first observed that tumor cells preferentially utilize glycolysis rather than OXPHOS for energy acquisition even under aerobic conditions, a phenomenon known as the ‘Warburg effect’ or aerobic glycolysis (77). Beyond tumor cells, immune cells also regulate their functional states through glycometabolic reprogramming. However, glucose depletion and lactic acid accumulation caused by tumor cells can significantly inhibit the metabolic adaptation and immune function of immune cells, thereby promoting immune escape (78). A deeper understanding of glucose metabolism between tumor cells and immune cells is therefore essential for devising metabolic interventions that can improve immunotherapy outcomes. Therefore, we summarized the reprogramming of glucose metabolism between OS and important immune cells in the immune microenvironment of OS (**Figure 1**).

#### 3.1.1 OS cells

Studies have indicated that aerobic glycolysis also serves as a metabolic modality through which OS cells acquire nutrients and sustain growth and proliferation (79). The metabolic shift from OXPHOS to aerobic glycolysis in OS cells may be associated with key enzymes in glucose metabolism. Hexokinase(HK) phosphorylates glucose into glucose-6-phosphate, serving as the first rate-limiting enzyme in the glycolytic process. Studies have found that HK2 is overexpressed in OS, meeting the energy demands of tumor cells by promoting glucose metabolism while simultaneously increasing lactic acid production, thereby affecting the pH of the TIME (80, 81).

Within OS cells, the expression of HK2 is regulated by miRNAs and lncRNAs. Liu et al. identified the glycolytic rate-limiting enzyme HK2 as a potential downstream target of miR-185. Their work shows that miR-185 inhibits glucose consumption and lactate production in OS cells. Conversely, its downregulation in OS promotes HK2 expression and enhances glycolysis, thereby accelerating tumor progression (82). The long non-coding RNA (lncRNA) Taurine Upregulated Gene 1 (TUG1) is considered an oncogene in OS. In OS cells, lncRNA TUG1 overexpression results in elevated HK2 protein levels, promoting glucose consumption and accelerating lactic acid production (83).

Phosphofructokinase-1 (PFK-1) catalyzes the conversion of fructose-6-phosphate to fructose-1,6-bisphosphate, serving as the second rate-limiting enzyme in glycolysis and the most critical enzyme regulating glycolytic flux. The COP9 signalosome subunit 3 (COPS3), implicated in various cancers for promoting proliferation and metastasis, is upregulated in OS. The upregulation of COPS3 enhances the glycolysis rate of OS cells via PFKFB3 regulation (84). But limited literature directly addresses the role of PFK-1 in glycolysis. Further research is needed to elucidate the PFKFB-PFK-1-aerobic glycolysis regulatory axis.

Pyruvate kinase (PK) catalyzes the final step of glycolysis, where phosphoenolpyruvate undergoes substrate-level phosphorylation to generate pyruvate, serving as the third rate-limiting enzyme in glycolysis. PK is encoded by two genes, PKLR and PKM, and is classified into PKL (liver-type isozyme), PKR (erythrocyte-type isozyme), PKM1 (muscle-type isozyme M1), and PKM2 (muscle-type isozyme M2) (85). Recent research indicates that overexpression of PKM2 is associated with chemotherapy resistance. Research has revealed that cancer stem cells (CSCs) exhibit significant resistance to cisplatin, while PKM2 shows marked overexpression in OS stem cells compared to non-CSCs. Knockdown of PKM2 restores the sensitivity of OS stem cells to cisplatin treatment, providing a novel potential therapeutic target for overcoming chemoresistance in OS (86).

OS also regulates glycolysis through the modulation of glucose transporters (GLUT). In mammals, glucose transport is mediated by GLUT on the cell membrane (87). Fan et al. found elevated expression levels of GLUT-1 in OS patients, with its expression positively correlated with tumor volume, degree of differentiation, lymph node metastasis, TNM staging, as well as cancer metastasis and recurrence (88). This may be due to GLUT-1 providing energy support for the growth and proliferation of OS cells.

#### 3.1.2 TAMs

Stimulation with LPS and IFN-γ induces a metabolic switch from OXPHOS to glycolysis in M1-type macrophages, thereby suppressing TCA cycle activity (89). By contrast, M2-type macrophages exhibit heightened OXPHOS, secrete immunosuppressive IL-10, and downregulate TNF-α production (90). The study by Chen et al. constructed a risk score model based on lactic acid metabolism-related prognostic genes SLC7A7 and CYP27A1, which could classify OS patients into high-risk and low-risk groups. Analyses showed significantly lower levels of both immune cell infiltration and immune activation in the high-risk group relative to the low-risk group. Further analysis identified a distinct macrophage composition between risk groups. The low-risk group was characterized by greater infiltration of M1-type TAMs, whereas the high-risk group had fewer macrophages overall, predominantly of the immunosuppressive M2 phenotype. However, the mechanism by which lactic acid affects TAM polarization in the TIME of OS remains unclear.

Another study has indicated that the metabolic activity of macrophages themselves may also influence the progression of OS. In OS patients, elevated PLEK expression correlates with prolonged overall survival and enhanced infiltration of immune cells, particularly TAMs and DCs. PLEK is enriched in macrophage populations exhibiting active glycolysis and OXPHOS pathways, suggesting that it may promote M1 phenotype conversion by modulating macrophage metabolic states, thereby enhancing antitumor functions (68). These studies have demonstrated that in osteosarcoma, the glucose metabolism characteristics of TAMs often exhibit glycolytic inhibition, thereby promoting the transformation to M2 type and enhancing immunosuppression.

#### 3.1.3 T cells

T cells in the TIME often experience disorders of glucose metabolism in various states. Specifically, due to glucose depletion and lactic acid accumulation in the tumor microenvironment, the glycolytic and oxidative phosphorylation activities of CD8+ T cells are inhibited, ultimately leading to T cell depletion, which is manifested as a reduction in the secretion of effector molecules and an increase in the expression of immunosuppressive factors (91). Conversely, Tregs demonstrate a metabolic shift toward OXPHOS, which facilitates their survival within the TIME (92). Studies indicate that the risk score of glycolysis-related genes in OS is significantly correlated with the degree of infiltration of CD8+ T cells and naïve CD4+ T cells, suggesting that glycolytic activity may modulate immune infiltration, thereby influencing growth of OS and patient prognosis (69). However, the glucose metabolism characteristics of T cells in the TIME of OS have not been directly expounded in the literature, which may require further verification.

Metabolic profiles of T cells also exhibit heterogeneity across different TIME contexts. OS pulmonary metastasis can induce pleural thickening and calcification, often accompanied by malignant pleural effusion (MPE). The enrichment level of immune cells in MPE is higher than that in primary tumors (PT). The study found that the proportion of CD8+ T cells in MPE was higher than in PT samples, but they still exhibited similar cytotoxicity and exhaustion characteristics. Further comparison of metabolic activity scores among T cell subsets in MPE and PT revealed that MPE-derived T cells showed increased activity in glycolysis, gluconeogenesis, pentose, and mannose metabolism, while OXPHOS activity decreased (70). This indicates that although metabolic reprogramming in metastatic lesions enhances the proliferation and survival capabilities of T cells, their immune activity remains suppressed.

#### 3.1.4 NK cells

Activated NK cells under normal conditions often rely on metabolic reprogramming to enhance glucose uptake and glycolytic activity, thereby sustaining effector functions such as proliferation, degranulation, and cytokine secretion (60). However, due to the competitive consumption of glucose by tumor cells and the large accumulation of the metabolic product lactic acid, the cytotoxic activity of NK cells has significantly decreased.

A prognostic model related to hypoxia and lactate metabolism, along with a risk score model developed by Chen et al. based on lactate metabolism-related prognostic genes, both revealed reduced NK cell infiltration in the TIME of high-risk group patients. This suggests that the metabolic characteristics of the tumor immune microenvironment may influence both the infiltration levels and functional status of NK cells (71). A study on OS immunotherapy utilized functionalized selenium nanoparticles (SeNPs) combined with metformin to treat NK92 cells, enhancing NK cell-mediated immunotherapeutic efficacy. Results demonstrated that this strategy significantly improved their cytotoxic efficacy against OS cells (93). However, the specific mechanism by which metformin enhances NK cell sensitization remains unclear and is likely associated with the modulation of glucose metabolism pathways.

### 3.2 Lipid metabolism

**Figure 2:**
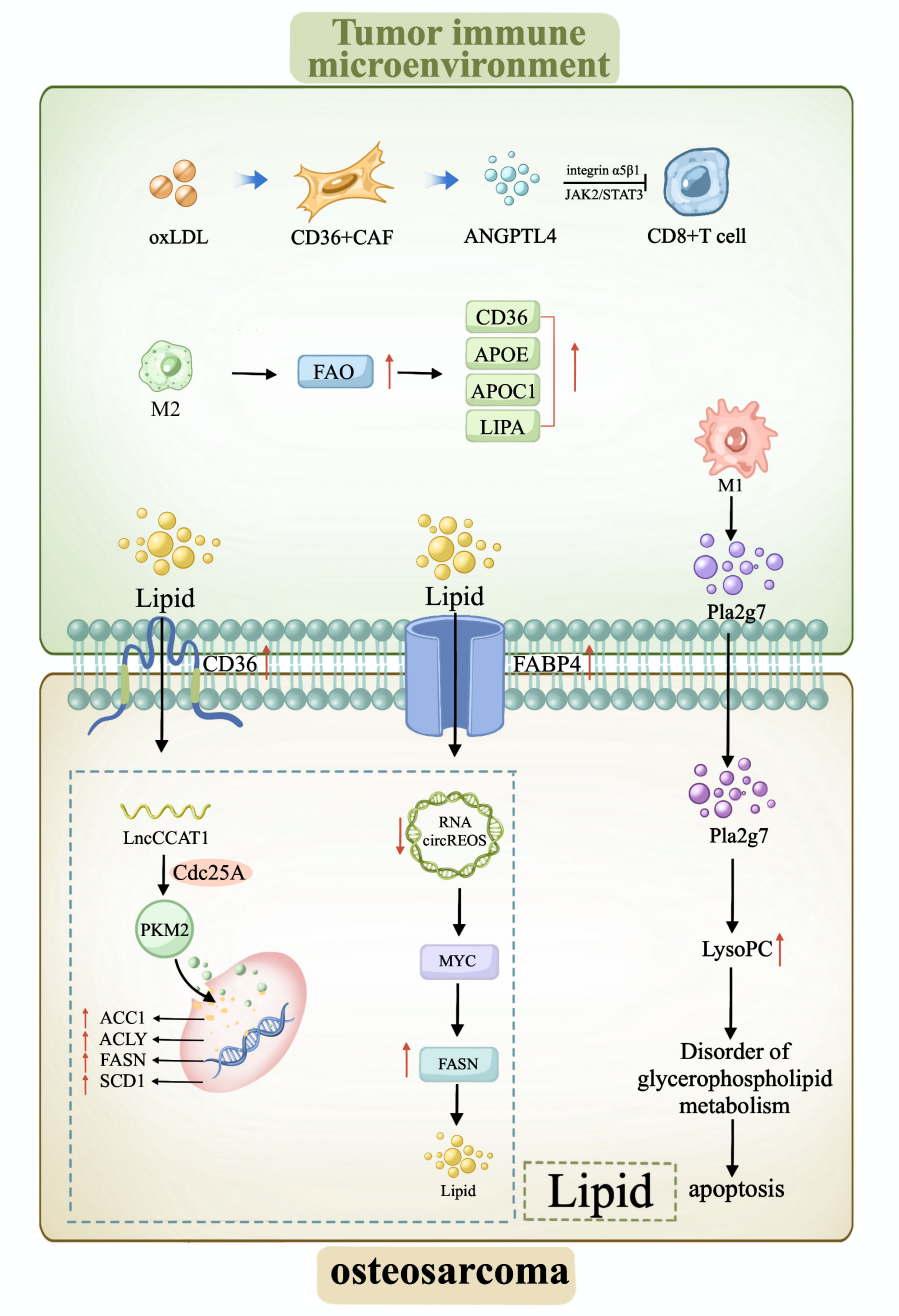
Reprogramming of lipid metabolism in OS and its TIME. Elevated expression of CD36 and FABP4 in OS cells promotes lipid uptake and synthesis, driving tumor progression. In OS, LncCCAT1 induces PKM2 nuclear translocation by recruiting Cdc25A, thereby upregulating the transcription of lipogenesis-related genes, including ACC1, ACLY, FASN, and SCD1, and promoting lipogenesis. The MYC-inhibiting circular RNA circREOS is downregulated in OS, leading to increased FASN expression and lipid accumulation. Lipid accumulation promotes the malignant progression of OS. Fatty acid oxidation (FAO) activity is also elevated in M2-type TAMs, which induces an immunosuppressive phenotype in TAMs. Elevated OxLDL in the TIME stimulates CD36+ CAFs to secrete ANGPTL4, which suppresses CD8+ T cell activity via the integrin α5β1-JAK2/STAT3 signaling pathway.

Growing evidence indicates that lipid metabolic reprogramming is highly prevalent in cancer. Lipogenesis is markedly upregulated in cancer to meet the increased demand for cell membrane biosynthesis. Lipid uptake and storage are also significantly enhanced in malignant tumors, thus targeting lipid metabolism regulatory pathways has emerged as a novel anticancer strategy (94). Biological processes such as FAO play important metabolic regulatory roles in enabling immune cells to adapt to nutrient scarcity within the TIME (95). OS cells and immune cells also interact by competing for nutrients and secreting metabolic substances, mediating immune escape (**Figure 2**).

#### 3.2.1 OS cells

Based on the expression profiles of lipid metabolism-related genes, Li et al. classified OS cells into C1 and C2 subtypes. The C1 subtype is characterized by enhanced biosynthesis of cholesterol and fatty acids, while the C2 subtype primarily involves steroid hormone biosynthesis. This classification could advance personalized and precision therapies for bone tumors and further highlights the potential of targeting lipid metabolism reprogramming as a therapeutic strategy (96).

Global lipidomic analysis of OS cells revealed that lipids such as diacylglycerol were significantly upregulated in metastatic OS cells (97). Elevated intracellular lipid content can be achieved through both endogenous and exogenous pathways. Further investigation revealed that, compared to normal cells, OS cells exhibit not only increased lipid levels but also enhanced endogenous lipid synthesis pathways (98). while In OS, LncCCAT1 induces PKM2 dephosphorylation and nuclear translocation by recruiting Cdc25A, thereby mediating phosphorylation of Sterol regulatory element-binding proteins(SREBP2) at the threonine 610 residue. This stabilizes SREBP2 and upregulates transcription of lipogenesis-related genes including ACC1, ACLY, FASN, and SCD1, promoting lipogenesis (99).

MYC is highly expressed in OS and has been demonstrated to serve as a prognostic biomarker and potential therapeutic target (16). Tong et al. discovered that downregulation of the MYC-inhibiting circular RNA circREOS in OS cells leads to increased fatty acid synthase (FASN) expression and lipid accumulation, thereby promoting malignant progression of OS (100). FADS2, a fatty acid desaturase, catalyzes polyunsaturated fatty acid synthesis and directly impacts lipid biosynthesis. Research indicates FADS2 is highly expressed in OS. The mechanism involves highly expressed circular RNA hsa_circ_0000073, which promotes lipid synthesis in OS by reducing miR-1184 expression and derepressing its inhibition of FADS2 (101).

Exogenous lipid uptake primarily relates to transport molecules on cell membranes. Transporters such as CD36, fatty acid transporter proteins, and fatty acid binding proteins (FABPs) facilitate the entry of exogenous fatty acids into cells, playing critical roles in cancer cell growth, metastasis, and EMT (102).

Studies have reported the correlation between overexpression of these transport molecules and poor prognosis in OS, as well as their role in metabolic reprogramming. Zhang et al. used 10 machine learning algorithms to screen for inflammasome genes most relevant to overall survival, among which CD36 was identified. *In vitro* experiments showed that knocking down CD36 significantly inhibited the proliferation, invasion, and migration capabilities of OS cells (103). However, the changes in fatty acid and lipid metabolism within OS following CD36 knockout have not been investigated, nor has the correlation between CD36-mediated lipid reprogramming and OS progression been elucidated. This emphasizes the importance of further research to explore the potential significance of targeting CD36 for the treatment of OS.

#### 3.2.2 TAMs

Lipid metabolism also plays a significant role in TAM polarization. TAMs, particularly M2-like macrophages with pro-tumor and angiogenic properties, rely on FAO for their metabolic processes. Studies have shown that genes associated with lipid metabolism are linked to the TIME and prognosis in OS patients (72). A single-cell RNA sequencing-based study identified distinct macrophage subpopulations within the OS immune microenvironment, among which the C06 macrophage subpopulation employed lipid metabolism to promote immunosuppression and tissue repair. This finding suggests an interplay between cellular function and metabolic reprogramming (12). In OS patients exhibiting poor response to chemotherapy, macrophages demonstrated significant enrichment and heightened activity in lipid metabolism-related pathways. Concurrently, key lipid metabolism molecules such as CD36, APOE, APOC1, and LIPA showed upregulated expression, indicating that heightened lipid metabolism may contribute to impaired immune function in these macrophages (73).

TAMs undergo metabolic reprogramming in lipid metabolism through multiple pathways. CD36, serving as a fatty acid receptor, facilitates the shift in TAMs from glycolysis to FAO for energy derivation, thereby enhancing their immunosuppressive function (104). DPP7, highly expressed in TAMs of patients with poor prognosis, enhances FAO metabolism by binding to and stabilizing CPT1A, a key enzyme in FAO, thereby inhibiting its ubiquitination-mediated degradation, which promotes M2 polarization of TAMs (105).

#### 3.2.3 T cells

Under glucose-deficient conditions, CD8+ T cells switch to utilizing fatty acids as an alternative energy source, relying on FAO to sustain survival, but their anti-tumor effects gradually diminish (106). Simultaneously, the activation of SREBPs in CD8+ T cells promotes the transcription of lipogenesis-related genes such as ACC1 and FASN (107).

Unlike CD8+ T cells, Tregs rely more on exogenous fatty acids within the TIME rather than endogenous lipid synthesis. Tregs can exacerbate tumor-associated immunosuppression by promoting SREBP1-dependent lipid metabolism to indirectly induce M2-like polarization of TAMs, while suppressing IFN-γ production in CD8+ T cells (108).

A recent study revealed that OS patients with a high cuproptosis-sphingolipid metabolism score exhibited adverse prognosis, strongly correlated with reduced CD8+ T cell infiltration (74). This suggests that cuproptosis-sphingolipid metabolism is involved in the recruitment of CD8+ T cells within the OS microenvironment. Therefore, targeting cuproptosis-sphingolipid metabolic pathways may enhance the efficacy of immunotherapies such as CAR-T against OS, although the precise mechanisms remain unclear.

#### 3.2.4 NK cells

Lipid metabolism not only generates energy to sustain NK cell survival but also promotes the biosynthesis of perforin and key mediators of tumor lysis (109). Within the TIME, elevated levels of free fatty acids can suppress mitochondrial metabolism in NK cells and impair the synthesis of anti-tumor immune factors IFN-γ and TNF-α. To adapt to the lipid-enriched microenvironment, NK cells may initiate lipid metabolic reprogramming, manifested by upregulation of peroxisome proliferator-activated receptor gamma and FABP4/5 expression, thus promoting lipid synthesis and storage to counteract exogenous lipotoxicity (110). Chen et al. found that in the TIME of OS, the cytotoxic activity of NK cells relies on significant enrichment in lipid metabolism and the TCA cycle pathway, which is consistent with the metabolic characteristics of NK cells in the general tumor microenvironment (12). This enhances, to some extent, the cytotoxicity, immune surveillance capabilities, and metabolic plasticity of NK cells. However, persistent or excessive lipid accumulation ultimately leads to functional inhibition of NK cells.

A study constructed a prognostic prediction model for OS based on arachidonic acid metabolism-related genes. Correlation analysis revealed that STOM exhibited the strongest positive correlation with NK cells, while PANX3 showed the highest negative correlation with central memory CD8+ T cells, suggesting that arachidonic acid metabolism is also associated with NK cell functionality (75). However, the specific mechanisms underlying lipid metabolism reprogramming in the immunosuppressive microenvironment of OS concerning NK cells require further research to be fully elucidated.

### 3.3 Amino acid metabolism

Beyond glucose and lipids, amino acids constitute a vital component supporting cellular proliferation, migration, and other biological processes. Recent studies have increasingly recognized the importance of amino acid metabolic reprogramming in tumor cells. Dysregulated amino acid metabolism promotes tumor aggressiveness and drug resistance (111). Therefore, targeting this process through strategies such as amino acid–degrading enzymes, transporter inhibitors, or biosynthesis pathway blockers holds significant therapeutic potential for cancer treatment (112).

**Figure 3:**
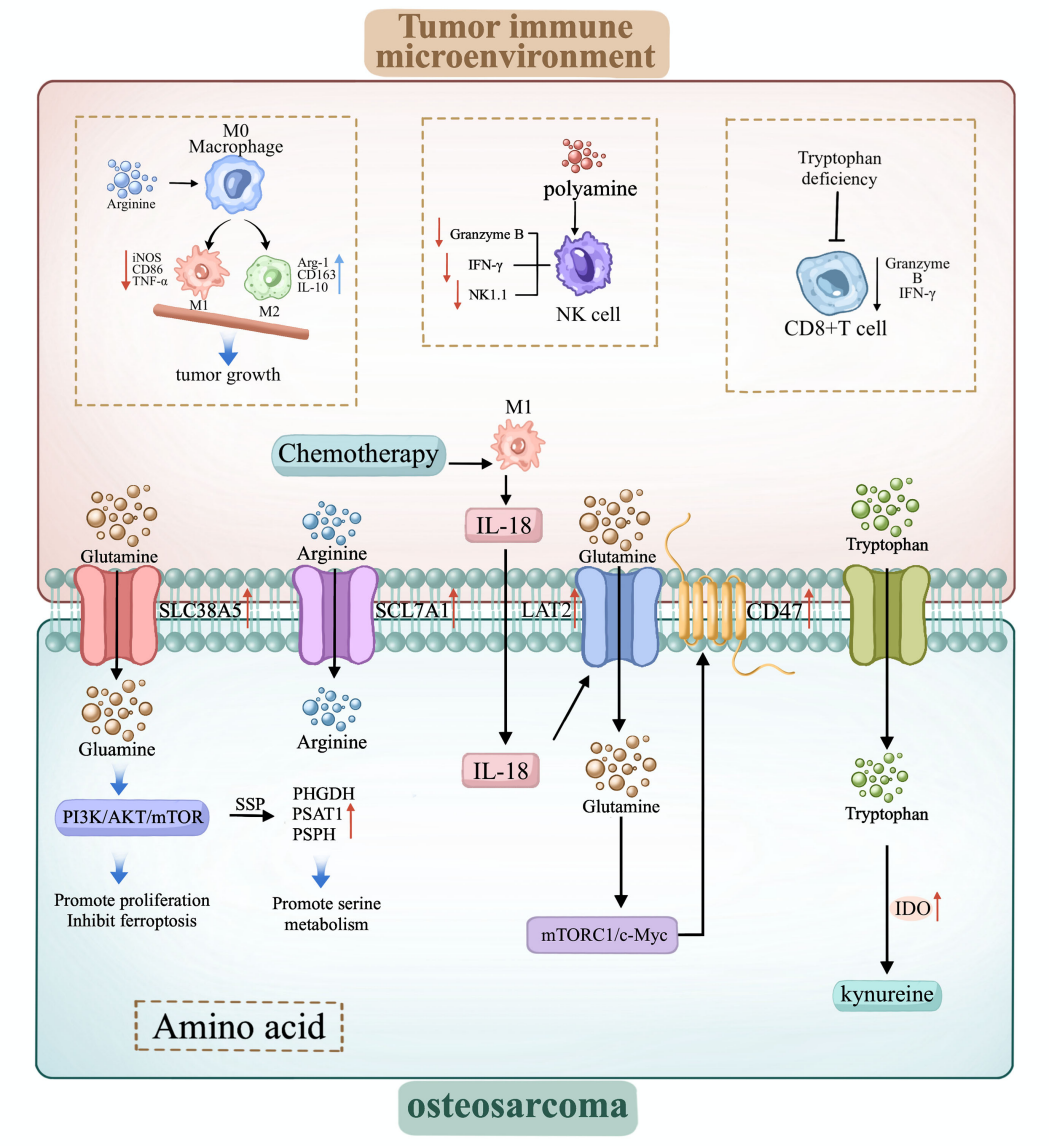
Reprogramming of amino acid metabolism in OS and its TIME. Increased expression of amino acid transporters SLC38A5 and SLC7A1 on the OS cell membrane enhances the uptake of glutamine and arginine, thereby supporting OS growth via activation of the PI3K/AKT/mTOR and SSP pathways, while concurrently depleting glutamine and arginine in the TIME and impairing immune cell function. Under chemotherapeutic conditions, increased expression of LAT2 and enhanced glutamine uptake activate the mTORC1/c-Myc pathway, ultimately upregulating CD47 expression on the cell membrane and suppressing immune cell function. Arginine metabolism in OS favors M2 polarization; accumulation of polyamines suppresses NK cell cytotoxicity. IDO-mediated kynurenine production depletes tryptophan, impairing CD8+ T cell effector function.

Amino acid metabolism exerts a profound influence on immune cell function and fate in OS by remodeling the TIME (**Figure 3**).

#### 3.3.1 OS cells

As the most abundant free amino acid in the body, glutamine sustains the TCA cycle by providing a carbon source. Glutamine catabolism proceeds through its conversion to glutamate by glutaminase (GLS), followed by transformation into α-ketoglutarate via glutamate dehydrogenase or transaminases, ultimately entering the TCA cycle (113). Ren et al. demonstrated that highly metastatic OS cells rely on glutamine as a crucial nutritional source for cellular proliferation both *in vivo* and *in vitro* (114). This might be related to the increase in glutamine transporters on the OS cell membrane. SLC38A5, which is responsible for transporting glutamine into cells, is highly expressed in osteosarcoma and correlates strongly with unfavorable patient prognosis. Upregulated SLC38A5 promotes malignant progression of OS and inhibits ferroptosis through glutamine-mediated PI3K/AKT/mTOR signaling pathway (115).

Serine is a neutral amino acid that enters cells via Na+-dependent transporters such as alanine-serine-cysteine transporter 1(ASCT1) (116). In tumor cells, the *de novo* serine synthase pathway (SSP) is frequently employed, utilizing the glycolytic intermediate 3-phosphoglycerate. Through sequentially catalyzed enzymatic reactions by PHGDH, phosphoserine aminotransferase (PSAT1), and phosphoserine phosphatase (PSPH), serine is ultimately synthesized. This pathway is essential for protein and nucleotide synthesis and contributes to the production of glycine and cysteine (117). Although there have been no reports on the impact of exogenous serine on OS proliferation and metastasis. Studies have revealed that PHGDH is highly expressed in OS and correlates with reduced survival rates, indicating heavy reliance on the SSP (118). While PHGDH inhibition attenuates OS cell proliferation, it fails to induce cell death because the resulting accumulation of S-adenosylmethionine/methionine triggers a compensatory activation of the mTOR survival pathway (119). Metabolomic analyses demonstrated that mTORC1 inhibition significantly reduced intracellular serine and glycine levels in OS cells. Following mTORC1 activation, the expression of key enzymes in the SSP, including PHGDH, PSAT1, and PSPH, is upregulated, thereby promoting serine metabolism. Given that serine and its metabolite glycine serve as crucial precursors for glutathione (GSH) synthesis, mTORC1 activation also enhances the antioxidant defense capacity of OS cells, strengthening their survival (120). Combined use of PHGDH inhibitors with non-rapamycin mTORC1 inhibitors (e.g., Perhexiline, ALPI3MT55) suppresses AKT and activates AMPK, leading to dephosphorylation and activation of transcription factor FOXO3. Activated FOXO3 translocates into the nucleus to transcriptionally upregulate the pro-apoptotic protein PUMA, ultimately synergistically inducing robust caspase-dependent apoptosis (119). This indicates that the regulation of serine metabolism within OS exhibits intricate complexity, making combination therapy a crucial treatment strategy.

#### 3.3.2 TAMs

TAMs frequently undergo reprogramming of multiple amino acid metabolic pathways, such as those involving arginine, tryptophan, and glutamine. M1-type macrophages predominantly express iNOS. iNOS catalyzes the conversion of arginine to NO and L-citrulline, where NO serves as a crucial pro-inflammatory and anti-tumor molecule. Conversely, M2-type TAMs predominantly express ARG1. In M2-type TAMs, arginine is primarily catabolized into urea and ornithine, facilitating tissue repair while promoting tumor cell proliferation (121). Concurrently, M2-type tumor-associated macrophages upregulate cationic amino acid transporter 1 and 2B expression, further exacerbating arginine depletion. This compromises the anti-tumor immune function of CD8+ T cells and ultimately leads to CD8+ T cell exhaustion (122). Indoleamine 2,3-dioxygenase (IDO) catalyzes the breakdown of tryptophan into kynurenine. IDO is highly expressed in M2-type TAMs, leading to increased tryptophan uptake and consumption, elevated kynurenine levels, and reshaping of the immunosuppressive microenvironment (123). Research on tryptophan in the OS microenvironment has primarily focused on OS cells and T cells, while the metabolic reprogramming of tryptophan in TAMs requires further investigation.

#### 3.3.3 T cells

Metabolic analysis of the OS immune microenvironment reveals that naïve T cells rely on amino acid metabolism to maintain their activation potential (12). Glutamine serves as the primary source of amino acids in activated CD8+ T cells. However, excessive glutamine uptake by tumor cells may lead to local microenvironmental glutamine deficiency, potentially compromising the anti-tumor response of CD8+ T cells (124). Simultaneously, elevated GSH levels in Tregs confer heightened resistance to ROS, influence serine uptake via ASCT1, and maintain FoxP3 expression to preserve their immunosuppressive function (125).

#### 3.3.4 NK cells

IL-2/IL-12-mediated activation of NK cell metabolism and function relies on c-Myc, with glutamine and other essential amino acids transported via SLC7A5 (such as leucine) being essential for sustaining c-Myc expression (126). Essential amino acids including leucine and arginine are also critical for NK cell activation. These amino acids trigger mTOR-dependent signaling cascades, thereby promoting NK cell proliferation, differentiation, and the secretion of anti-tumor immune factors IFN-γ and TNF-α, ultimately enhancing NK cell cytotoxicity (60). The activity of amino acid metabolism-related enzymes such as arginase or GLS is often elevated in tumor cells, resulting in depletion of arginine or glutamine within the TIME. This consequently suppresses the metabolism and function of NK cells. while.

## 4 Molecular mechanisms of immunosuppression induced by metabolic reprogramming

**Figure 4:**
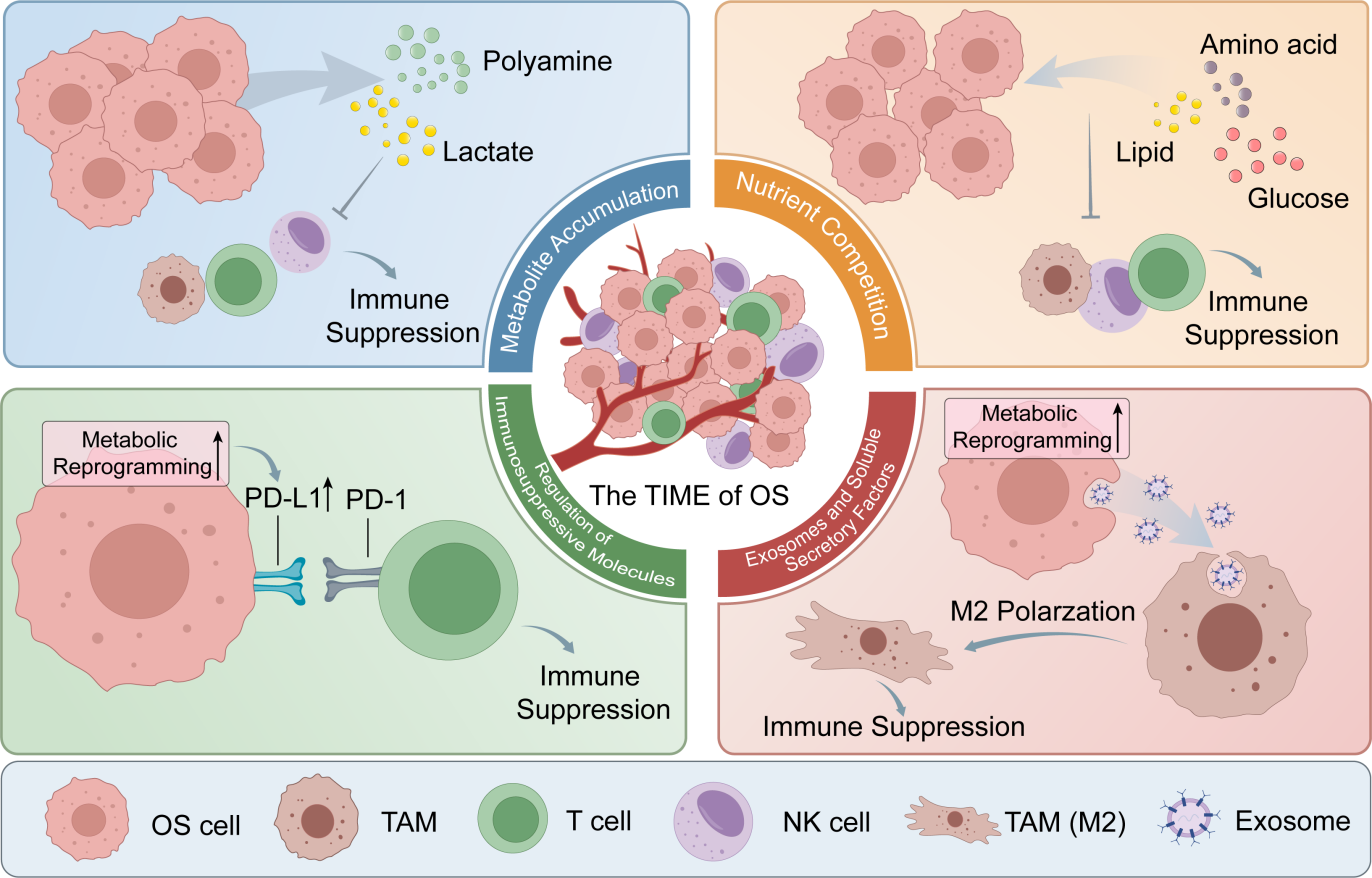
Schematic illustration of immunosuppression driven by metabolic reprogramming in OS. OS cells remodel the tumor microenvironment and suppress antitumor immunity through four interconnected mechanisms: Metabolite accumulation—OS cells secrete lactate, polyamines, and other metabolites that directly impair immune cell function; Nutrient competition—enhanced uptake of glucose, lipids, and amino acids by OS cells depletes these nutrients in the microenvironment, restricting metabolic adaptation and effector functions of immune cells; Upregulation of immunosuppressive molecules—metabolic reprogramming of OS cells upregulates immune checkpoint molecules such as PD-L1, which directly inhibit antitumor activity by engaging PD-1 on T cells; Exosomes and soluble factors—metabolic reprogramming promotes the secretion of exosomes and soluble factors from OS cells, driving macrophage polarization toward the immunosuppressive M2 phenotype. Collectively, these mechanisms establish the immunosuppressive microenvironment in osteosarcoma.

Based on current preclinical research, the functional status and metabolic characteristics of immune cells in the OS microenvironment are closely associated with metabolic reprogramming of OS cells, which can regulate immune cell function through multiple metabolic pathways, primarily including: (1) Competitively consuming key nutrients in the microenvironment, thereby impairing the metabolic adaptability of immune cells and leading to functional inhibition; (2) Producing harmful metabolites such as lactate and polyamines, directly damaging the survival and effector functions of immune cells; (3) Inducing the expression of immunosuppressive molecules (e.g., PD-L1) through metabolic reprogramming, directly inhibiting the anti-tumor activity of immune cells; and (4) Promoting the secretion of exosomes, delivering metabolism-related signals to immune cells and remotely regulating their functional status. These intertwined metabolic-immune interaction mechanisms collectively form the foundation of the immunosuppressive microenvironment in OS. These studies based on OS in mice will systematically elaborate on the regulatory role of metabolic reprogramming in the OS immune microenvironment from four aspects: nutrient competition, metabolite accumulation, regulation of immunosuppressive molecules, and exosome-mediated signaling (**Figure 4**).

### 4.1 Nutrient competition

Nutrient competition is a critical mechanism by which metabolic reprogramming mediates immunosuppression in the TIME. In various tumors, due to competitive glucose uptake by tumor cells, glucose levels in the microenvironment are significantly reduced, thereby hindering the glucose metabolic reprogramming of TAMs, T cells, and NK cells and inhibiting their anti-tumor activity (76). Studies have shown that in various tumors, the activity of amino acid metabolism-related enzymes such as arginase and glutaminase is often elevated in tumor cells, leading to depletion of arginine or glutamine in the microenvironment, which in turn inhibits the metabolism and function of NK cells. Specifically, glutamine deficiency significantly impairs the ability of NK cells to produce IFN-γ and express granzyme B, while arginine deficiency impairs anti-tumor function by downregulating the expression of NK cell receptors (including NKp46, NKp30, and the ζ-chain) and reducing IFN-γ production (126, 127). Additionally, arginine depletion can lead to T cell dysfunction and even apoptosis, and its degradation also causes reduced CD3 expression, impairing T cell responsiveness (128, 129).

In OS, the nutrient competition described above is also prevalent, although existing research mainly focuses on amino acid metabolism. OS cells with high SLC7A1 expression may deplete arginine in the TIME through enhanced arginine uptake, thereby stimulating the activity of the arginine biosynthesis pathway in TAMs and promoting their polarization toward the M2 phenotype (130). Furthermore, OS cells may overexpress IDO, catalyzing the oxidation of tryptophan to kynurenine, leading to excessive tryptophan depletion in the microenvironment and thereby suppressing tryptophan-dependent T cell function and activity (131). These findings indicate that targeted OS cell metabolic reprogramming can reverse the metabolic abnormalities of immune cells by reducing the competitive pressure for nutrients in the TIME, thereby enhancing their immune activity.

### 4.2 Metabolite accumulation

Metabolic reprogramming of tumor cells leads to the abnormal accumulation of various metabolites, which can impair immune cell function through direct or indirect mechanisms.

#### 4.2.1 Glycolytic metabolites

Enhanced tumor glycolysis results in substantial lactate accumulation. Lactate can suppress IFN-γ production in NK cells by downregulating nuclear factor of activated T cells (NFAT), thereby impairing their anti-tumor immune function (92). The accumulation of lactate also creates a low-pH acidic microenvironment. This acidic environment not only directly impairs the metabolic activity of NK cells but also downregulates the expression of the critical activation receptor NKp46 on their surface, further compromising the ability of NK cells to recognize and eliminate tumor cells (132). Lactate and hydrogen ions have also been identified as major factors driving TAM polarization toward the M2 phenotype in the OS microenvironment (133). However, the mechanisms by which metabolites derived from glycolytic reprogramming lead to immune cell dysfunction in OS have not been fully investigated.

#### 4.2.2 Lipid metabolites

In OS, abnormalities in lipid metabolites are also involved in the regulation of immunosuppression. Immunohistochemical analysis of clinical OS samples from non-responders to PD-1 immunotherapy revealed elevated levels of oxidized low-density lipoprotein (OxLDL) within the TIME. Single-cell sequencing in patients with poor response to PD-1 immunotherapy revealed significant enrichment of CD36+ cancer-associated fibroblasts (CAFs). Further investigation demonstrated that after CD36+ CAFs uptake OxLDL, they interact with CD8+ T cells, ultimately inducing CD8+ T cell exhaustion and functional impairment, thereby mediating the immunosuppressive tumor microenvironment and resistance to PD-1 immunotherapy (134).

#### 4.2.3 Amino acid metabolites

In the TIME, polyamines, as naturally occurring immunosuppressive metabolites, can reduce the cytolytic activity of NK cells, thereby promoting tumor immune escape (135). Specifically, In colon cancer, polyamines can suppress the expression of NK1.1 receptors, perforin, and IFN-γ on NK cells, weakening their ability to recognize and kill tumor cells (136). In OS, the risk model constructed based on polyamine metabolism-related genes indicates that patients in the low-risk group have a better prognosis, and polyamine levels in the TIME may be reduced, thereby alleviating the immunosuppressive effects on NK cells. This may be one of the underlying mechanisms contributing to the favorable prognosis in this patient subgroup (137).

### 4.3 Regulation of immunosuppressive molecules

In addition to nutrient competition and metabolite accumulation, metabolic reprogramming of tumor cells can also directly or indirectly impair immune cell function by regulating the expression of immunosuppressive molecules.

In various tumors, bidirectional regulatory relationships exist between immunosuppressive molecules and metabolic pathways. For example, the PD-1-PD-L1 axis can stimulate FAO in CD8+ T cells through the STAT3 pathway while inhibiting glycolysis and IFN-γ production, thereby weakening the anti-tumor function of T cells (138). Furthermore, tumor cells can utilize the Fas-FasL axis to activate acid sphingomyelinase in CD8+ T cells, non-apoptotically blocking calcium channels in T lymphocytes through ceramide to suppress T cell activation and mediate immune escape (139).

In OS, metabolic reprogramming is also involved in the regulation of immunosuppressive molecules. OS can directly regulate the expression of various immunomodulatory factors through aerobic glycolysis, affecting the proliferation, activity, and differentiation of immune cells (140). For instance, under glucose-deprived conditions, OS enhances its own survival by significantly upregulating NUCB2 expression. Concurrently, NUCB2 stabilizes NUCKS1 protein, activating the CXCL8/PD-L1 axis to directly inhibit the cytotoxicity of CD8+ T cells and mediate immune escape (141). However, the relationship between other metabolic pathways and the expression of immunosuppressive factors in OS cells remains unclear.

### 4.4 Exosomes and soluble secretory factors

Soluble secretory factors also participate in the metabolic processes of OS cells and the interactions between OS cells and immune cells. In OS cells, endogenous lactate can upregulate the expression of macrophage migration inhibitory factor (MIF) via histone H3K9 lactylation, thereby promoting TAM polarization toward the M2 phenotype, specifically manifested by upregulated expression of anti-inflammatory markers ARG1, CD163, and IL-10, alongside downregulated expression of pro-inflammatory markers iNOS, CD86, and TNF-α. Furthermore, OS cell-derived MIF can suppress the production of CXCL9 and CXCL10 by macrophages, thereby downregulating granzyme B and IFN-γ in CD8+ T cells and ultimately promoting tumor immune escape (67). Secretory factors can also be produced by immune cells to regulate OS cell metabolism, thereby promoting malignant progression of OS. Studies have shown that chemotherapy can induce TAM polarization toward the M1 phenotype and promote IL-18 secretion. IL-18 acting on OS cells can upregulate the expression of L-amino acid transporter 2 (LAT2), thereby enhancing glutamine and leucine uptake by OS cells, leading to glutamine depletion in the TIME. Concurrently, this process activates the mTORC1/c-Myc signaling pathway, ultimately resulting in CD47 upregulation and facilitating immune escape (142).

Additionally, exosomes are key mediators of tumor regulation of macrophage polarization. In immunotherapy-resistant OS patients, CD36+ CAFs accelerate exogenous lipid uptake and regulate CD8+ T cell function by secreting ANGPTL4 protein (134). Furthermore, studies have found that IDO1 overexpression in OS can regulate the secretion of exosomal miRNA hsa-miR-23a-3p, promoting OS progression. Although this study did not identify the specific immune cells targeted, the target genes of this exosomal miRNA are associated with dendritic cell differentiation and T cell maturation, suggesting that OS may regulate immune cell function through IDO1 overexpression-promoted exosomal miRNA secretion (143).

## 5 Potential therapeutic strategies

Since metabolic reprogramming is a key factor in immunosuppression, focusing on this process offers a viable way to improve the effectiveness of immunotherapy in OS. Therefore, interventions targeting OS metabolic reprogramming and reshaping immune cell metabolism have emerged as promising anti-tumor strategies. Concurrently, metabolic reprogramming has been found to enhance the efficacy of immunotherapy. For patients exhibiting a poor response to immunotherapy, combining therapies that target metabolic reprogramming with immunotherapy is considered a promising future direction. This section will explore therapeutic approaches combining metabolic reprogramming targeting tumor cells and their immune microenvironment with immunotherapy. A summary of relevant studies on anti-metabolic reprogramming drugs and their impact on immunotherapy, categorized by targeted cell type, is presented in **Table 3**.

**Table 3:** Summary of relevant studies on anti-metabolic reprogramming drugs and immunotherapy.

Metabolic type	Drug/strategy	Mechanism of action	Experimental approach	Impact on immunotherapy	Ref
Glucose Metabolism	SGLT2 Inhibitor (Canagliflozin)	Mechanism may involve inhibition of AKT phosphorylation	*In Vivo* Murine Experiment	Enhance efficacy of anti-PD-1 therapy, promoting CD4+ and CD8+ T cell infiltration in OS.	(144)
	Metformin	Alter metabolism of CD11b+ cells, shifting from high OXPHOS to high glycolysis	*In Vivo* Murine Experiment	Reduce PMN-MDSCs in TIME; promote TAMs repolarization from M2 to M1 phenotype; alleviate immunosuppression and enhance PD-1 blockade efficacy.	(145)
	TW80-SeNPs + Metformin	Unspecified	*In Vitro* Murine experiments	Enhances the cytotoxic effects of NK cells against OS cells.	(93)
	SHK@Mn-TiO_2_ nano-platform (combined with SDT)	SHK@Mn-TiO_2_: Directly inhibits PKM2; Released Mn²^+^: Downregulates HK-2 via cGAS-STING pathway; SHK: Reduces oxygen consumption and HIF-1α, decreasing lactic acid accumulation	*In Vivo* Murine Experiment	Reverses SDT-induced immunosuppressive TIME: Elevates IFN-γ and TNF-α levels; Promotes CTL infiltration and DC maturation; Reduces Tregs and MDSCs.	(146)
Amino Acid Metabolism	L-arginine + α-PD-L1 antibody	Replenish depleted arginine in the TIME to support immune cell functions such as T cells	*In Vivo* Murine Experiment	In combination with α-PD-L1 antibody, it significantly suppresses OS growth and prolongs survival.	(147)

### 5.1 Targeting tumor metabolism

Given that tumor metabolic reprogramming can inhibit immune cell activity through multiple pathways, including competitive consumption of nutrients in the TIME, secretion of harmful metabolites, upregulation of immunosuppressive molecule expression, and release of exosomes, targeting tumor metabolic reprogramming is expected to become an important adjuvant strategy for reversing the immunosuppressive microenvironment and overcoming immunotherapy resistance.

Targeting metabolic reprogramming has also shown promising prospects in preclinical studies of OS treatment. For example, inhibitors of sodium-glucose cotransporter 2 (SGLT2) can suppress the high expression of SGLT2 in OS cells of mouse, thereby reshaping tumor glucose metabolism and further weakening tumor growth and angiogenic activity. Combined immunotherapy (such as anti-PD-1 therapy) can further promote the infiltration of CD4+ and CD8+ T cells in OS (144). In recent years, the novel nano-platform SHK@Mn-TiO2 has further expanded the strategy of targeted metabolic intervention. This platform effectively inhibits PKM2 expression in OS, and the released Mn2+ ions downregulate the key glycolytic enzyme HK-2, achieving dual inhibition of glycolysis. Notably, this platform also reduces intracellular oxygen consumption, significantly decreases HIF-1α levels, reverses the hypoxic TIME, and diminishes lactic acid accumulation. Traditional sonodynamic therapy (SDT) often induces an immunosuppressive TIME, whereas SHK@Mn-TiO2 reverses this phenomenon, manifesting as elevated extracellular levels of IFN-γ and TNF-α, promoting CTL infiltration and DC maturation, while reducing the proportions of Tregs and MDSCs, thereby establishing long-term immune memory and laying a solid foundation for subsequent immunotherapy to achieve significant anti-tumor immunotherapeutic efficacy (146). Similarly, the IDO inhibitor NLG919 enhances CD8+ T cell activity, activates anti-tumor immunity, and potentiates the efficacy of platinum-based drugs against OS by inhibiting tryptophan metabolism in OS cells (131). These findings suggest that targeting metabolic reprogramming in OS cells, as a therapeutic strategy, may fundamentally reverse the formation of the immunosuppressive TIME and enhance the response of OS to immunotherapy. However, this therapeutic strategy still faces translational challenges, including insufficient immune responses within the TIME and inadequate targeting specificity.

### 5.2 Targeting immune cell metabolism

However, therapeutic strategies targeting only tumor cell metabolism are often insufficient. Because the physical properties of the tumor microenvironment and its metabolites continuously inhibit the immune function of CD8+ T cells and M1-type TAMs, while immunosuppressive cells such as CSCs, MDSCs, and M2-type TAMs further reshape the immunosuppressive metabolic microenvironment, this is why metabolic reprogramming drugs alone are difficult to enter clinical trials. Correcting metabolic disorders in immune cells, restoring metabolic homeostasis in the tumor microenvironment, and enhancing their metabolic adaptability are also potential strategies to effectively improve immune responses in the tumor microenvironment, thereby enhancing the response of OS to immunotherapy.

Previous studies have shown that metformin enhances the efficacy of PD-1 blockade by reducing tumor hypoxia. Specifically, metformin treatment reduces basal respiration in CD11b+ cells including MDSCs and TAMs within OS, shifting their metabolic phenotype from high OXPHOS to high glycolysis, while simultaneously downregulating fatty acid uptake in TAMs and inhibiting their FAO process. This metabolic reprogramming ultimately decreases the proportion of PMN-MDSCs in the TIME and reprograms TAMs from the M2 phenotype to the M1 phenotype, thereby alleviating the immunosuppressive state in OS (145). Another study has revealed the combination of functionalized selenium nanoparticles with metformin in NK92 cells significantly enhances NK cell-mediated immunotherapeutic efficacy, manifested as markedly improved cytotoxic activity against OS cells (93). However, the specific mechanism by which metformin enhances NK cell sensitization remains unclear and may be associated with the modulation of glucose metabolism pathways.

On the other hand, direct supplementation of deficient amino acids within the TIME has also been shown to effectively restore immune cell function. L-arginine, a key amino acid for T cell activation, is often depleted in the OS microenvironment due to competitive uptake by tumor cells. Studies have shown that exogenous supplementation of L-arginine combined with α-PD-L1 antibody significantly inhibits OS growth *in vivo* and prolongs survival (147). This strategy restores the metabolic adaptability of T cells and enhances their anti-tumor immune response, suggesting that amino acid supplementation may serve as an important adjunct strategy for immunotherapy.

In summary, targeting immune cell metabolism can reshape anti-tumor immune responses by blocking immunosuppressive metabolic crosstalk or supplementing essential nutrients, providing new intervention strategies for OS immunotherapy.

## 6 Challenges and future directions

### 6.1 Enhancing specificity to mitigate off-target effects

The majority of metabolic inhibitors have poor selectivity for tumor cells, which is a significant translational obstacle. Since metabolic pathways such as glycolysis, fatty acid metabolism, and glutamine metabolism are crucial not only in immune effector cells but also in other cells, systemic metabolic inhibition might suppress tumor metabolism while inadvertently impairing immune cell function. This “metabolic off-target” effect can negates the synergistic potential of combined treatment while causing systemic toxicity (148). This lack of specificity might be a primary reason why targeted metabolic reprogramming drugs in combination with immunotherapy have yet to be widely adopted in clinical practice.

Enhancing selectivity and reducing off-target risks are crucial, with precise delivery strategies and identification of metabolic dependencies specific to OS being key directions. Delivery systems based on nanotechnology can improve the solubility, stability, and tumor accumulation efficiency of metabolic inhibitors while minimizing systemic exposure to mitigate side effects (149). For instance, in triple-negative breast cancer models, engineered lipid nanoparticles modified with a human receptor tyrosine kinase-like orphan receptor 1 antibody (ROR1 Ab) achieved precise tumor targeting and visual tracking via NIR-II fluorescence imaging, effectively reversing the immunosuppressive tumor microenvironment and promoting CD8+ T cell infiltration (150). Although this study was not specifically aimed at OS, its delivery concept and immuno-metabolic synergy provide a valuable translational pathway for bone tumor research.

### 6.2 Overcoming resistance to sustain therapeutic response

The ignored metabolic and immunological heterogeneity of metastatic lesions, which differ significantly from initial tumors, is a major contributing factor to therapy resistance. In metastatic OS lung lesions, IDO1+ immunosuppressive cells are enriched and directly interact with PD-1+ cytotoxic T cells. This interaction is associated with poor patient prognosis, and its mechanism involves IDO1-driven tryptophan metabolism, which depletes essential amino acids and releases accumulated kynurenine into the TIME, thereby suppressing T cell effector function. Combining IDO inhibitors with immune checkpoint blockade therapy may represent a promising strategy for certain subsets of OS patients (151). Unfortunately, early results from a Phase II clinical trial evaluating the combination of the PD-1 antibody pembrolizumab and the IDO1 inhibitor epacadostat in advanced sarcomas showed that this regimen was associated with limited anti-tumor responses. This outcome may be attributable to the significant heterogeneity in the metabolic-immune microenvironment among different OS patients and between primary and metastatic/recurrent lesions (152). Therefore, utilizing emerging technologies such as single-cell sequencing and spatial transcriptomics to perform metabolic-immune profiling of the OS microenvironment can facilitate the identification of key metabolic pathway dependencies and critical immune cells, thereby enabling stratified and dynamically adjusted combination therapy strategies. This precision profiling may not only improve initial response rates but also help delay or reverse treatment-related resistance. Existing studies using single-cell transcriptomic analysis have revealed that OS lung metastases are predominantly infiltrated by M2-type TAMs enriched in lipid metabolism pathways, which are closely associated with an immunosuppressive phenotype, while the anti-tumor activity of NK cells concurrently depends on the co-activation of the TCA cycle and lipid metabolism (12, 67). These findings suggest that precision reprogramming strategies targeting the metabolic characteristics of different immune cell subsets could serve as effective approaches to enhance anti-tumor immunity.

Although specialized research into metabolic-immunotherapy targeting OS metastases is lacking, preliminary exploration in other solid tumors offers an insightful reference. A Phase II clinical trial (MARIO-3) targeting first-line metastatic triple-negative breast cancer patients employed the highly selective PI3K-γ inhibitor Eganelisib in combination with immune checkpoint inhibitors and chemotherapy. This approach successfully induced systemic immune activation and improved clinical outcomes by reprogramming TAMs from the M2 to the M1 phenotype (153). Despite certain limitations, including a single-arm design that cannot distinguish the independent contributions of individual drugs and treatment interruptions in some patients due to adverse events, both of which constrain the precision of mechanistic attribution and efficacy evaluation, the study provides a cutting−edge approach for OS by integrating immunotherapy with metabolism−reprogramming−targeted therapy. Specifically, intervening at key metabolic nodes to reshape the immune microenvironment may help overcome the current response bottlenecks in immunotherapy.

However, directly applying these strategies to OS faces multiple challenges. Current studies primarily rely on primary OS models, while responses to combined treatments in metastatic or secondary OS lack systemic research. Considering that metastatic lesions might have distinct metabolic dependencies and microenvironment characteristics, the efficacy of metabolic intervention in metastatic OS, drug accumulation at lesion sites, and long-term systemic toxicity remain to be elucidated. Thus, developing reliable animal models capable of simulating OS metastatic metabolic characteristics is a critical prerequisite for advancing translational applications in this field. Moreover, the metabolic dependencies of OS metastatic sites have yet to be systematically analyzed, lacking clear intervention targets. Finally, the dynamic evolution of metabolomic profiles and immune cell function following combined treatment interventions is still unclear. Future research must employ multi-omics and dynamic monitoring techniques to map the metabolic-immune spatiotemporal landscape in patient-derived metastatic samples, establish animal models replicating the microenvironment characteristics, and systematically analyze dynamic response processes and molecular regulatory networks post-combined treatment. This will provide a theoretical basis and translational foundation for the development of precise combined therapy strategies.

## 7 Conclusion

OS, as a primary malignant bone tumor, frequently occurs in pediatric patients and adolescents. It is characterized by high rates of metastasis and recurrence, resulting in a poor prognosis. Although neoadjuvant chemotherapy combined with surgical intervention can improve outcomes for patients with localized disease, survival rates for metastatic and recurrent OS remain suboptimal, highlighting the urgent need to explore novel therapeutic strategies. In recent years, with in-depth research into the intrinsic mechanisms of tumor immunology, novel immunotherapeutic approaches, such as cancer vaccines, cellular immunotherapy, and immune checkpoint inhibitors, have been developed and progressively applied in clinical practice. However, due to the uniquely immunosuppressive microenvironment in OS, particularly pronounced in metastatic lesions, the overall efficacy of current immunotherapies remains suboptimal. Elucidating the mechanisms underlying the formation of the immunosuppressive microenvironment in OS and those conferring resistance to immunotherapy will provide a critical foundation for optimizing immunotherapeutic strategies in this disease. Recent evidence indicates that metabolic reprogramming plays a crucial role in shaping the immunosuppressive tumor microenvironment during OS progression. This review delineates the metabolic reprogramming characteristics of glucose, lipids, and amino acid metabolism in OS and its microenvironment. It summarizes how these alterations dynamically regulate the functional states of immune cells, such as tumor-associated macrophages, T cells, and NK cells, through nutrient competition, metabolite accumulation, and exosome-mediated molecular transfer, thus facilitating immune escape. Combining metabolic inhibitors with immune checkpoint blockade, CAR-T cell therapy, or conventional chemotherapy has demonstrated synergistic anti-tumor effects in multiple studies, offering novel strategies to overcome immunotherapy resistance in OS. However, current evidence remains primarily confined to preclinical studies. Issues such as insufficient targeting specificity and significant systemic toxicity observed with certain metabolic inhibitors indicate that the clinical benefits of combining them with immunotherapy remain to be further validated. Future approaches may leverage nanoparticle-mediated delivery for tumor-targeted transport or utilize techniques like sonodynamic therapy to precisely focus on tumor sites while minimizing damage to surrounding healthy tissues. These strategies offer a promising means to achieve more precise modulation of the TIME of OS, thereby improving the safety and efficacy of combination therapy involving metabolic reprogramming inhibitors and immunotherapy.

## References

[1] Ritter J Bielack SS . Osteosarcoma. Ann Oncol. (2010) 21:vii320–5. doi: 10.1093/annonc/mdq276. PMID: 20943636

[2] Zhou Y Yang D Yang Q Lv X Huang W Zhou Z . Single-cell RNA landscape of intratumoral heterogeneity and immunosuppressive microenvironment in advanced osteosarcoma. Nat Commun. (2020) 11:6322. doi: 10.1038/s41467-020-20059-6. PMID: 33303760 PMC7730477

[3] Beird HC Bielack SS Flanagan AM Gill J Heymann D Janeway KA . Osteosarcoma. Nat Rev Dis Primer. (2022) 8:77. doi: 10.1038/s41572-022-00409-y. PMID: 36481668

[4] Yu S Yao X . Advances on immunotherapy for osteosarcoma. Mol Cancer. (2024) 23:192. doi: 10.1186/s12943-024-02105-9. PMID: 39245737 PMC11382402

[5] Meltzer PS Helman LJ . New horizons in the treatment of osteosarcoma. N Engl J Med. (2021) 385:2066–76. doi: 10.1056/NEJMra2103423. PMID: 34818481

[6] Marina NM Smeland S Bielack SS Bernstein M Jovic G Krailo MD . Comparison of MAPIE versus MAP in patients with a poor response to preoperative chemotherapy for newly diagnosed high-grade osteosarcoma (EURAMOS-1): an open-label, international, randomised controlled trial. Lancet Oncol. (2016) 17:1396–408. doi: 10.1016/S1470-2045(16)30214-5. PMID: 27569442 PMC5052459

[7] Kager L Zoubek A Pötschger U Kastner U Flege S Kempf-Bielack B . Primary metastatic osteosarcoma: Presentation and outcome of patients treated on neoadjuvant cooperative osteosarcoma study group protocols. J Clin Oncol. (2003) 21:2011–8. doi: 10.1200/JCO.2003.08.132. PMID: 12743156

[8] Weiss SA Djureinovic D Jessel S Krykbaeva I Zhang L Jilaveanu L . A phase I study of APX005M and Cabiralizumab with or without Nivolumab in patients with melanoma, kidney cancer, or non–small cell lung cancer resistant to anti-PD-1/PD-L1. Clin Cancer Res. (2021) 27:4757–67. doi: 10.1158/1078-0432.CCR-21-0903. PMID: 34140403 PMC9236708

[9] Elsallab M Levine BL Wayne AS Abou-El-Enein M . CAR T-cell product performance in haematological Malignancies before and after marketing authorisation. Lancet Oncol. (2020) 21:e104–16. doi: 10.1016/S1470-2045(19)30729-6. PMID: 32007196 PMC7841982

[10] Xie L Liang X Xu J Sun X Liu K Sun K . Exploratory study of an anti-PD-L1/TGF-β antibody, TQB2858, in patients with refractory or recurrent osteosarcoma and alveolar soft part sarcoma: a report from Chinese sarcoma study group (TQB2858-Ib-02). BMC Cancer. (2023) 23:868. doi: 10.1186/s12885-023-11390-4. PMID: 37715133 PMC10503089

[11] Davis KL Fox E Merchant MS Reid JM Kudgus RA Liu X . Nivolumab in children and young adults with relapsed or refractory solid tumours or lymphoma (ADVL1412): a multicentre, open-label, single-arm, phase 1–2 trial. Lancet Oncol. (2020) 21:541–50. doi: 10.1016/S1470-2045(20)30023-1. PMID: 32192573 PMC7255545

[12] Chen J Cui N He SH Xia CY Li WQ . Single-cell transcriptomics reveals metabolic remodeling and functional specialization in the immune microenvironment of bone tumors. J Transl Med. (2025) 23:554. doi: 10.1186/s12967-025-06346-0. PMID: 40380260 PMC12084933

[13] Zhu Y Yan W Tong L Yang J Ge S Fan J . Metabolic reprogramming: A crucial contributor to anticancer drug resistance. MedComm. (2025) 6:e70358. doi: 10.1002/mco2.70358. PMID: 40919131 PMC12413565

[14] Liu S Zhang X Wang W Li X Sun X Zhao Y . Metabolic reprogramming and therapeutic resistance in primary and metastatic breast cancer. Mol Cancer. (2024) 23:261. doi: 10.1186/s12943-024-02165-x. PMID: 39574178 PMC11580516

[15] Lv B Wang Y Ma D Cheng W Liu J Yong T . Immunotherapy: Reshape the tumor immune microenvironment. Front Immunol. (2022) 13:844142. doi: 10.3389/fimmu.2022.844142. PMID: 35874717 PMC9299092

[16] Wu C Gong S Duan Y Deng C Kallendrusch S Berninghausen L . A tumor microenvironment-based prognostic index for osteosarcoma. J BioMed Sci. (2023) 30:23. doi: 10.1186/s12929-023-00917-3. PMID: 37055822 PMC10099847

[17] Orrapin S Moonmuang S Udomruk S Yongpitakwattana P Pruksakorn D Chaiyawat P . Unlocking the tumor-immune microenvironment in osteosarcoma: Insights into the immune landscape and mechanisms. Front Immunol. (2024) 15:1394284. doi: 10.3389/fimmu.2024.1394284. PMID: 39359731 PMC11444963

[18] Cersosimo F Lonardi S Bernardini G Telfer B Mandelli GE Santucci A . Tumor-associated macrophages in osteosarcoma: From mechanisms to therapy. Int J Mol Sci. (2020) 21:5207. doi: 10.3390/ijms21155207. PMID: 32717819 PMC7432207

[19] Taylor AM Sheng J Ng PKS Harder JM Kumar P Ahn JY . Immunosuppressive tumor microenvironment of osteosarcoma. Cancer (Basel). (2023) 17:2117. doi: 10.3390/cancers17132117. PMID: 40647416 PMC12248827

[20] Zhang GZ Wu ZL Li CY Ren EH Yuan WH Deng YJ . Development of a machine learning-based autophagy-related lncRNA signature to improve prognosis prediction in osteosarcoma patients. Front Mol Biosci. (2021) 8:615084. doi: 10.3389/fmolb.2021.615084. PMID: 34095215 PMC8176230

[21] Yang H Zhao L Zhang Y Li F . A comprehensive analysis of immune infiltration in the tumor microenvironment of osteosarcoma. Cancer Med. (2021) 10:5696–711. doi: 10.1002/cam4.4117. PMID: 34258887 PMC8366103

[22] Pan R Pan F Zeng Z Lei S Yang Y Yang Y . A novel immune cell signature for predicting osteosarcoma prognosis and guiding therapy. Front Immunol. (2022) 13:1017120. doi: 10.3389/fimmu.2022.1017120. PMID: 36189307 PMC9515362

[23] Yang B Su Z Chen G Zeng Z Tan J Wu G . Identification of prognostic biomarkers associated with metastasis and immune infiltration in osteosarcoma. Oncol Lett. (2021) 21:180. doi: 10.3892/ol.2021.12441. PMID: 33574919 PMC7816295

[24] Ma Y Yang F Yang J Wang K Hu J Wu Q . The multifaceted role of macrophages in kidney physiology and diseases. Front Immunol. (2025) 16:1642525. doi: 10.3389/fimmu.2025.1642525. PMID: 41050663 PMC12490994

[25] Huang Q Liang X Ren T Huang Y Zhang H Yu Y . The role of tumor-associated macrophages in osteosarcoma progression – therapeutic implications. Cell Oncol. (2021) 44:525–39. doi: 10.1007/s13402-021-00598-w. PMID: 33788151 PMC12980758

[26] Christofides A Strauss L Yeo A Cao C Charest A Boussiotis VA . The complex role of tumor-infiltrating macrophages. Nat Immunol. (2022) 23:1148–56. doi: 10.1038/s41590-022-01267-2. PMID: 35879449 PMC10754321

[27] He L Jhong JH Chen Q Huang KY Strittmatter K Kreuzer J . Global characterization of macrophage polarization mechanisms and identification of M2-type polarization inhibitors. Cell Rep. (2021) 37:109955. doi: 10.1016/j.celrep.2021.109955. PMID: 34731634 PMC8783961

[28] Zheng X Mansouri S Krager A Grimminger F Seeger W Pullamsetti SS . Metabolism in tumour-associated macrophages: a quid pro quo with the tumour microenvironment. Eur Respir Rev. (2020) 29:200134. doi: 10.1183/16000617.0134-2020. PMID: 33004525 PMC9488699

[29] Lai G Zhao X Chen Y Xie T Su Z Lin J . The origin and polarization of macrophages and their role in the formation of the pre-metastatic niche in osteosarcoma. Int Immunopharmacol. (2025) 150:114260. doi: 10.1016/j.intimp.2025.114260. PMID: 39938167

[30] Han Q Shi H Liu F . CD163 + M2-type tumor-associated macrophage support the suppression of tumor-infiltrating T cells in osteosarcoma. Int Immunopharmacol. (2016) 34:101–6. doi: 10.1016/j.intimp.2016.01.023. PMID: 26938675

[31] Dhupkar P Gordon N Stewart J Kleinerman ES . Anti‐PD‐1 therapy redirects macrophages from an M2 to an M1 phenotype inducing regression of OS lung metastases. Cancer Med. (2018) 7:2654–64. doi: 10.1002/cam4.1518. PMID: 29733528 PMC6010882

[32] Su Y Zhou Y Sun Y Wang YL Yin J Huang Y . Macrophage-derived CCL18 promotes osteosarcoma proliferation and migration by upregulating the expression of UCA1. J Mol Med. (2019) 97:49–61. doi: 10.1007/s00109-018-1711-0. PMID: 30426155

[33] Yang S Jia J Wang F Wang Y Fang Y Yang Y . Targeting neutrophils: Mechanism and advances in cancer therapy. Clin Transl Med. (2024) 14:e1599. doi: 10.1002/ctm2.1599. PMID: 38450975 PMC10918741

[34] Mollinedo F . Neutrophil degranulation, plasticity, and cancer metastasis. Trends Immunol. (2019) 40:228–42. doi: 10.1016/j.it.2019.01.006. PMID: 30777721

[35] Jaillon S Ponzetta A Di Mitri D Santoni A Bonecchi R Mantovani A . Neutrophil diversity and plasticity in tumour progression and therapy. Nat Rev Cancer. (2020) 20:485–503. doi: 10.1038/s41568-020-0281-y. PMID: 32694624

[36] Xia M Han Y Sun L Li D Zhu C Li D . The role of neutrophils in osteosarcoma: Insights from laboratory to clinic. Front Immunol. (2024) 15:1490712. doi: 10.3389/fimmu.2024.1490712. PMID: 39582869 PMC11582048

[37] Xu J Shi Q Zeng F Ren T Wei R Tang X . Neutrophil elastase promotes low molecular weight cyclin E1 formation to accelerate osteosarcoma proliferation. Front Immunol. (2025) 16:1647913. doi: 10.3389/fimmu.2025.1647913. PMID: 40959067 PMC12434046

[38] Papayannopoulos V . Neutrophil extracellular traps in immunity and disease. Nat Rev Immunol. (2018) 18:134–47. doi: 10.1038/nri.2017.105. PMID: 28990587

[39] Chu D Huang R Shi J Xu R Wei D . NETs-related genes predict prognosis and are correlated with the immune microenvironment in osteosarcoma. Front Oncol. (2025) 15:1551074. doi: 10.3389/fonc.2025.1551074. PMID: 40276062 PMC12018237

[40] Tang H Xie J Du YX Tan ZJ Liang ZT . Osteosarcoma neutrophil extracellular trap network-associated gene recurrence and metastasis model. J Cancer Res Clin Oncol. (2024) 150:48. doi: 10.1007/s00432-023-05577-2. PMID: 38285218 PMC10824883

[41] Lin Y Tang H Teng H Feng W Li F Liu S . Development and validation of neutrophil extracellular traps-derived signature to predict the prognosis for osteosarcoma patients. Int Immunopharmacol. (2024) 127:111364. doi: 10.1016/j.intimp.2023.111364. PMID: 38101221

[42] Moon CY Belabed M Park MD Mattiuz R Puleston D Merad M . Dendritic cell maturation in cancer. Nat Rev Cancer. (2025) 25:225–48. doi: 10.1038/s41568-024-00787-3. PMID: 39920276 PMC11954679

[43] Collin M Bigley V . Human dendritic cell subsets: An update. Immunology. (2018) 154:3–20. doi: 10.1111/imm.12888. PMID: 29313948 PMC5904714

[44] Liu Y Feng W Dai Y Bao M Yuan Z He M . Single-cell transcriptomics reveals the complexity of the tumor microenvironment of treatment-naive osteosarcoma. Front Oncol. (2021) 11:709210. doi: 10.3389/fonc.2021.709210. PMID: 34367994 PMC8335545

[45] Kawano M Itonaga I Iwasaki T Tsuchiya H Tsumura H . Anti-TGF-β antibody combined with dendritic cells produce antitumor effects in osteosarcoma. Clin Orthop. (2012) 470:2288–94. doi: 10.1007/s11999-012-2299-2. PMID: 22415727 PMC3392369

[46] Kawano M Tanaka K Itonaga I Iwasaki T Miyazaki M Ikeda S . Dendritic cells combined with anti-GITR antibody produce antitumor effects in osteosarcoma. Oncol Rep. (2015) 34:1995–2001. doi: 10.3892/or.2015.4161. PMID: 26239052

[47] Li K Shi H Zhang B Ou X Ma Q Chen Y . Myeloid-derived suppressor cells as immunosuppressive regulators and therapeutic targets in cancer. Signal Transd Targ Ther. (2021) 6:362. doi: 10.1038/s41392-021-00670-9. PMID: 34620838 PMC8497485

[48] Wu Y Yi M Niu M Mei Q Wu K . Myeloid-derived suppressor cells: An emerging target for anticancer immunotherapy. Mol Cancer. (2022) 21:184. doi: 10.1186/s12943-022-01657-y. PMID: 36163047 PMC9513992

[49] Jiang K Li J Zhang J Wang L Zhang Q Ge J . SDF-1/CXCR4 axis facilitates myeloid-derived suppressor cells accumulation in osteosarcoma microenvironment and blunts the response to anti-PD-1 therapy. Int Immunopharmacol. (2019) 75:105818. doi: 10.1016/j.intimp.2019.105818. PMID: 31437795

[50] Marvel D Gabrilovich DI . Myeloid-derived suppressor cells in the tumor microenvironment: Expect the unexpected. J Clin Invest. (2015) 125:3356–64. doi: 10.1172/JCI80005. PMID: 26168215 PMC4588239

[51] Masopust D Schenkel JM . The integration of T cell migration, differentiation and function. Nat Rev Immunol. (2013) 13:309–20. doi: 10.1038/nri3442. PMID: 23598650

[52] Franco F Jaccard A Romero P Yu YR Ho PC . Metabolic and epigenetic regulation of T-cell exhaustion. Nat Metab. (2020) 2:1001–12. doi: 10.1038/s42255-020-00280-9. PMID: 32958939

[53] Odorizzi PM Pauken KE Paley MA Sharpe A Wherry EJ . Genetic absence of PD-1 promotes accumulation of terminally differentiated exhausted CD8+ T cells. J Exp Med. (2015) 212:1125–37. doi: 10.1084/jem.20142237. PMID: 26034050 PMC4493417

[54] Bengsch B Johnson AL Kurachi M Odorizzi PM Pauken KE Attanasio J . Bioenergetic insufficiencies due to metabolic alterations regulated by the inhibitory receptor PD-1 are an early driver of CD8 + T cell exhaustion. Immunity. (2016) 45:358–73. doi: 10.1016/j.immuni.2016.07.008. PMID: 27496729 PMC4988919

[55] Gao W Zhou J Ji B . Evidence of interleukin 21 reduction in osteosarcoma patients due to PD-1/PD-L1-mediated suppression of follicular helper T cell functionality. DNA Cell Biol. (2017) 36:794–800. doi: 10.1089/dna.2017.3669. PMID: 28650673

[56] Liang H Cui M Tu J Chen X . Advancements in osteosarcoma management: integrating immune microenvironment insights with immunotherapeutic strategies. Front Cell Dev Biol. (2024) 12:1394339. doi: 10.3389/fcell.2024.1394339. PMID: 38915446 PMC11194413

[57] Sundara YT Kostine M Cleven AHG Bovée JVMG Schilham MW Cleton-Jansen AM . Increased PD-L1 and T-cell infiltration in the presence of HLA class I expression in metastatic high-grade osteosarcoma: a rationale for T-cell-based immunotherapy. Cancer Immunol Immunother. (2017) 66:119–28. doi: 10.1007/s00262-016-1925-3. PMID: 27853827 PMC5222929

[58] Park JA Cheung NKV . Promise and challenges of T cell immunotherapy for osteosarcoma. Int J Mol Sci. (2023) 24:12520. doi: 10.3390/ijms241512520. PMID: 37569894 PMC10419531

[59] Chiossone L Dumas PY Vienne M Vivier E . Natural killer cells and other innate lymphoid cells in cancer. Nat Rev Immunol. (2018) 18:671–88. doi: 10.1038/s41577-018-0061-z. PMID: 30209347

[60] Terrén I Orrantia A Vitallé J Zenarruzabeitia O Borrego F . NK cell metabolism and tumor microenvironment. Front Immunol. (2019) 10:2278. doi: 10.3389/fimmu.2019.02278. PMID: 31616440 PMC6769035

[61] Campbell KS Hasegawa J . Natural killer cell biology: an update and future directions. J Allergy Clin Immunol. (2013) 132:536–44. doi: 10.1016/j.jaci.2013.07.006. PMID: 23906377 PMC3775709

[62] Yang X Zhang W Xu P . NK cell and macrophages confer prognosis and reflect immune status in osteosarcoma. J Cell Biochem. (2019) 120:8792–7. doi: 10.1002/jcb.28167. PMID: 30556159

[63] Cho MM Song L Quamine AE Szewc F Shi L Ebben JD . CD155 blockade enhances allogeneic natural killer cell-mediated antitumor response against osteosarcoma. J Immunother Cancer. (2025) 13:e008755. doi: 10.1136/jitc-2023-008755. PMID: 40234092 PMC12001373

[64] Ogiwara Y Nakagawa M Nakatani F Uemura Y Zhang R Kudo-Saito C . Blocking FSTL1 boosts NK immunity in treatment of osteosarcoma. Cancer Lett. (2022) 537:215690. doi: 10.1016/j.canlet.2022.215690. PMID: 35439537

[65] Zhang M Chen L Li Y Kong D . PD-L1/PD-1 axis serves an important role in natural killer cell-induced cytotoxicity in osteosarcoma. Oncol Rep. (2019) 42:2049–56. doi: 10.3892/or.2019.7299. PMID: 31485666

[66] Zhu T Han J Yang L Cai Z Sun W Hua Y . Immune microenvironment in osteosarcoma: components, therapeutic strategies and clinical applications. Front Immunol. (2022) 13:907550. doi: 10.3389/fimmu.2022.907550. PMID: 35720360 PMC9198725

[67] Chen W Liao Y Yao H Zou Y Fang J Zhang J . Multiomics integration analysis identifies tumor cell-derived MIF as a therapeutic target and potentiates anti-PD-1 therapy in osteosarcoma. J Immunother Cancer. (2025) 13:e011091. doi: 10.1136/jitc-2024-011091. PMID: 40846326 PMC12374663

[68] Zou Y Kang J Zhu S Ren X Li Z Niu J . The osteosarcoma immune microenvironment in progression: PLEK as a prognostic biomarker and therapeutic target. Front Immunol. (2025) 16:1651858. doi: 10.3389/fimmu.2025.1651858. PMID: 40895569 PMC12394983

[69] Gui H Wang S Li B . Glycolysis-related gene signatures and the functional role of P4HA1 in osteosarcoma prognosis. Exp Cell Res. (2025) 447:114492. doi: 10.1016/j.yexcr.2025.114492. PMID: 40023306

[70] Zhang Z Ji W Huang J Zhang Y Zhou Y Zhang J . Characterization of the tumour microenvironment phenotypes in Malignant tissues and pleural effusion from advanced osteoblastic osteosarcoma patients. Clin Transl Med. (2022) 12:e1072. doi: 10.1002/ctm2.1072. PMID: 36305631 PMC9615475

[71] Wang Y Wang X Liu Y Xu J Zhu J Zheng Y . A novel hypoxia- and lactate metabolism-related prognostic signature to characterize the immune landscape and predict immunotherapy response in osteosarcoma. Front Immunol. (2024) 15:1467052. doi: 10.3389/fimmu.2024.1467052. PMID: 39569192 PMC11576178

[72] Qian H Lei T Hu Y Lei P . Expression of lipid-metabolism genes is correlated with immune microenvironment and predicts prognosis in osteosarcoma. Front Cell Dev Biol. (2021) 9:673827. doi: 10.3389/fcell.2021.673827. PMID: 33937273 PMC8085431

[73] Lin Z Wu Z Luo W . Bulk and single-cell sequencing identified a prognostic model based on the macrophage and lipid metabolism related signatures for osteosarcoma patients. Heliyon. (2024) 10:e26091. doi: 10.1016/j.heliyon.2024.e26091. PMID: 38404899 PMC10884844

[74] Li Q Fang J Liu K Luo P Wang X . Multi-omic validation of the cuproptosis-sphingolipid metabolism network: modulating the immune landscape in osteosarcoma. Front Immunol. (2024) 15:1424806. doi: 10.3389/fimmu.2024.1424806. PMID: 38983852 PMC11231095

[75] Wang Y Hsu P Hu H Lin F Wei X . Role of arachidonic acid metabolism in osteosarcoma prognosis by integrating WGCNA and bioinformatics analysis. BMC Cancer. (2025) 25:445. doi: 10.1186/s12885-024-13278-3. PMID: 40075313 PMC11905593

[76] Borde S Matosevic S . Metabolic adaptation of NK cell activity and behavior in tumors: challenges and therapeutic opportunities. Trends Pharmacol Sci. (2023) 44:832–48. doi: 10.1016/j.tips.2023.08.009. PMID: 37770314

[77] Liberti MV Locasale JW . The Warburg effect: how does it benefit cancer cells? Trends Biochem Sci. (2016) 41:211–8. doi: 10.1016/j.tibs.2015.12.001. PMID: 26778478 PMC4783224

[78] Andryszkiewicz W Gąsiorowska J Kübler M Kublińska K Pałkiewicz A Wiatkowski A . Glucose metabolism and tumor microenvironment: mechanistic insights and therapeutic implications. Int J Mol Sci. (2025) 26:1879. doi: 10.3390/ijms26051879. PMID: 40076506 PMC11900028

[79] Giang AH Raymond T Brookes P De Mesy Bentley K Schwarz E O’Keefe R . Mitochondrial dysfunction and permeability transition in osteosarcoma cells showing the Warburg effect. J Biol Chem. (2013) 288:33303–11. doi: 10.1074/jbc.M113.507129. PMID: 24100035 PMC3829176

[80] Sun L Wang P Zhang Z Zhang K Xu Z Li S . MicroRNA-615 functions as a tumor suppressor in osteosarcoma through the suppression of HK2. Oncol Lett. (2020) 20:1. doi: 10.3892/ol.2020.12089. PMID: 32968448 PMC7500052

[81] Zhuo B Li Y Li Z Qin H Sun Q Zhang F . PI3K/Akt signaling mediated Hexokinase-2 expression inhibits cell apoptosis and promotes tumor growth in pediatric osteosarcoma. Biochem Biophys Res Commun. (2015) 464:401–6. doi: 10.1016/j.bbrc.2015.06.092. PMID: 26116768

[82] Liu C Cai L Li H . miR-185 regulates the growth of osteosarcoma cells via targeting Hexokinase 2. Mol Med Rep. (2019) 20:2774–82. doi: 10.3892/mmr.2019.10534. PMID: 31524259 PMC6691194

[83] Han X Yang Y Sun Y Qin L Yang Y . LncRNA TUG1 affects cell viability by regulating glycolysis in osteosarcoma cells. Gene. (2018) 674:87–92. doi: 10.1016/j.gene.2018.06.085. PMID: 29960067

[84] Zhang F Li Q Zhang Y Li N Rao M Li S . COPS3 inhibition promotes cell proliferation blockage and anoikis via regulating PFKFB3 in osteosarcoma cancer cells. Eur J Pharmacol. (2023) 951:175799. doi: 10.1016/j.ejphar.2023.175799. PMID: 37201626

[85] Dayton TL Jacks T Vander Heiden MG . PKM 2, cancer metabolism, and the road ahead. EMBO Rep. (2016) 17:1721–30. doi: 10.15252/embr.201643300. PMID: 27856534 PMC5283597

[86] Shang D Wu J Guo L Xu Y Liu L Lu J . Metformin increases sensitivity of osteosarcoma stem cells to cisplatin by inhibiting expression of PKM2. Int J Oncol. (2017) 50:1848–56. doi: 10.3892/ijo.2017.3950. PMID: 28393220

[87] Klip A De Bock K Bilan PJ Richter EA . Transcellular barriers to glucose delivery in the body. Annu Rev Physiol. (2024) 86:149–73. doi: 10.1146/annurev-physiol-042022-031657. PMID: 38345907

[88] Fan J Mei J Zhang MZ Yuan F Li SZ Yu GR . Clinicopathological significance of glucose transporter protein-1 overexpression in human osteosarcoma. Oncol Lett. (2017) 14:2439–45. doi: 10.3892/ol.2017.6437. PMID: 28781680 PMC5530187

[89] Pantel A Teixeira A Haddad E Wood EG Steinman RM Longhi MP . Direct type I IFN but not MDA5/TLR3 activation of dendritic cells is required for maturation and metabolic shift to glycolysis after Poly IC stimulation. PloS Biol. (2014) 12:e1001759. doi: 10.1371/journal.pbio.1001759. PMID: 24409099 PMC3883643

[90] Mills CD Kincaid K Alt JM Heilman MJ Hill AM . M-1/M-2 macrophages and the Th1/Th2 paradigm. J Immunol. (2000) 164:6166–73. doi: 10.4049/jimmunol.164.12.6166. PMID: 10843666

[91] Chang CH Qiu J O’Sullivan D Buck MD Noguchi T Curtis JD . Metabolic competition in the tumor microenvironment is a driver of cancer progression. Cell. (2015) 162:1229–41. doi: 10.1016/j.cell.2015.08.016. PMID: 26321679 PMC4864363

[92] Brand A Singer K Koehl GE Kolitzus M Schoenhammer G Thiel A . LDHA-associated lactic acid production blunts tumor immunosurveillance by T and NK cells. Cell Metab. (2016) 24:657–71. doi: 10.1016/j.cmet.2016.08.011. PMID: 27641098

[93] Du Y Zhang Z Yang Y Liu T Chen T Li X . Highly active selenium nanotherapeutics combined with metformin to achieve synergistic sensitizing effect on NK cells for osteosarcoma therapy. Nanophotonics. (2022) 11:5101–11. doi: 10.1515/nanoph-2022-0289. PMID: 39634308 PMC11501141

[94] Liu Q Luo Q Halim A Song G . Targeting lipid metabolism of cancer cells: a promising therapeutic strategy for cancer. Cancer Lett. (2017) 401:39–45. doi: 10.1016/j.canlet.2017.05.002. PMID: 28527945

[95] Zhu L Zhu X Wu Y . Effects of glucose metabolism, lipid metabolism, and glutamine metabolism on tumor microenvironment and clinical implications. Biomolecules. (2022) 12:580. doi: 10.3390/biom12040580. PMID: 35454171 PMC9028125

[96] Li S Zheng Z Wang B . Machine learning survival prediction using tumor lipid metabolism genes for osteosarcoma. Sci Rep. (2024) 14:12934. doi: 10.1038/s41598-024-63736-y. PMID: 38839983 PMC11153634

[97] Roy J Dibaeinia P Fan TM Sinha S Das A . Global analysis of osteosarcoma lipidomes reveal altered lipid profiles in metastatic versus nonmetastatic cells. J Lipid Res. (2019) 60:375–87. doi: 10.1194/jlr.M088559. PMID: 30504231 PMC6358301

[98] Yin Z Shen G Fan M Zheng P . Lipid metabolic reprogramming and associated ferroptosis in osteosarcoma: from molecular mechanisms to potential targets. J Bone Oncol. (2025) 51:100660. doi: 10.1016/j.jbo.2025.100660. PMID: 39958756 PMC11830322

[99] Pu F Liu J Jing D Chen F Huang X Shi D . LncCCAT1 interaction protein PKM2 upregulates SREBP2 phosphorylation to promote osteosarcoma tumorigenesis by enhancing the Warburg effect and lipogenesis. Int J Oncol. (2022) 60:44. doi: 10.3892/ijo.2022.5334. PMID: 35244192 PMC8923656

[100] Tong W Wang S He C Li A Nie J Zuo W . CircREOS suppresses lipid synthesis and osteosarcoma progression through inhibiting HuR-mediated MYC activation. J Cancer. (2023) 14:916–26. doi: 10.7150/jca.83106. PMID: 37151387 PMC10158517

[101] Ren Z Wang S Li B Huang H Zhang H Yang Z . Hsa_circ_0000073 promotes lipid synthesis of osteosarcoma through hsa-miR-1184/ FADS2 pathway. Cell Signal. (2023) 110:110829. doi: 10.1016/j.cellsig.2023.110829. PMID: 37506860

[102] Broadfield LA Pane AA Talebi A Swinnen JV Fendt SM . Lipid metabolism in cancer: new perspectives and emerging mechanisms. Dev Cell. (2021) 56:1363–93. doi: 10.1016/j.devcel.2021.04.013. PMID: 33945792

[103] Zhang N Han Y Cao H Wang Q . Inflammasome‐related gene signatures as prognostic biomarkers in osteosarcoma. J Cell Mol Med. (2024) 28:e18286. doi: 10.1111/jcmm.18286. PMID: 38742843 PMC11092527

[104] Qin H Xiao A Lu Q Li Y Luo X Zheng E . The fatty acid receptor CD36 promotes macrophage infiltration via p110γ signaling to stimulate metastasis. J Adv Res. (2025) 74:237–53. doi: 10.1016/j.jare.2024.10.006. PMID: 39419288 PMC12302359

[105] Chang J Niu Y Zhou S Zhu W Zhang Z Xiu H . DPP7 promotes fatty acid β-oxidation in tumor-associated macrophages and determines immunosuppressive microenvironment in colorectal cancer. Int J Biol Sci. (2025) 21:6305–25. doi: 10.7150/ijbs.117909. PMID: 41208881 PMC12594597

[106] Tang Y Chen Z Zuo Q Kang Y . Regulation of CD8+ T cells by lipid metabolism in cancer progression. Cell Mol Immunol. (2024) 21:1215–30. doi: 10.1038/s41423-024-01224-z. PMID: 39402302 PMC11527989

[107] Kidani Y Elsaesser H Hock MB Vergnes L Williams KJ Argus JP . Sterol regulatory element–binding proteins are essential for the metabolic programming of effector T cells and adaptive immunity. Nat Immunol. (2013) 14:489–99. doi: 10.1038/ni.2570. PMID: 23563690 PMC3652626

[108] Liu C Chikina M Deshpande R Menk AV Wang T Tabib T . Treg cells promote the SREBP1-dependent metabolic fitness of tumor-promoting macrophages via repression of CD8+ T cell-derived interferon-γ. Immunity. (2019) 51:381–397.e6. doi: 10.1016/j.immuni.2019.06.017. PMID: 31350177 PMC6703933

[109] Ping Y Fan Q Zhang Y . Modulating lipid metabolism improves tumor immunotherapy. J Immunother Cancer. (2025) 13:e010824. doi: 10.1136/jitc-2024-010824. PMID: 39904563 PMC11795363

[110] Kobayashi T Lam PY Jiang H Bednarska K Gloury R Murigneux V . Increased lipid metabolism impairs NK cell function and mediates adaptation to the lymphoma environment. Blood. (2020) 136:3004–17. doi: 10.1182/blood.2020005602. PMID: 32818230

[111] Wei Z Liu X Cheng C Yu W Yi P . Metabolism of amino acids in cancer. Front Cell Dev Biol. (2021) 8:603837. doi: 10.3389/fcell.2020.603837. PMID: 33511116 PMC7835483

[112] Ge M Xu Y Cui L Huang E Liu Z Yin K . Targeting amino acid in tumor therapy. Front Oncol. (2025) 15:1582116. doi: 10.3389/fonc.2025.1582116. PMID: 40535116 PMC12174139

[113] Zhang J Pavlova NN Thompson CB . Cancer cell metabolism: the essential role of the nonessential amino acid, glutamine. EMBO J. (2017) 36:1302–15. doi: 10.15252/embj.201696151. PMID: 28420743 PMC5430235

[114] Ren L Ruiz-Rodado V Dowdy T Huang S Issaq SH Beck J . Glutaminase-1 (GLS1) inhibition limits metastatic progression in osteosarcoma. Cancer Metab. (2020) 8:4. doi: 10.1186/s40170-020-0209-8. PMID: 32158544 PMC7057558

[115] Huang X Xia K Wei Z Liu W Wei Z Guo W . SLC38A5 suppresses ferroptosis through glutamine-mediated activation of the PI3K/AKT/mTOR signaling in osteosarcoma. J Transl Med. (2024) 22:1004. doi: 10.1186/s12967-024-05803-6. PMID: 39511570 PMC11542360

[116] Riscal R Schrepfer E Arena G Cissé MY Bellvert F Heuillet M . Chromatin-bound MDM2 regulates serine metabolism and redox homeostasis independently of p53. Mol Cell. (2016) 62:890–902. doi: 10.1016/j.molcel.2016.04.033. PMID: 27264869

[117] Locasale JW . Serine, glycine and one-carbon units: cancer metabolism in full circle. Nat Rev Cancer. (2013) 13:572–83. doi: 10.1038/nrc3557. PMID: 23822983 PMC3806315

[118] Rathore R Caldwell KE Schutt C Brashears CB Prudner BC Ehrhardt WR . Metabolic compensation activates pro-survival mTORC1 signaling upon 3-phosphoglycerate dehydrogenase inhibition in osteosarcoma. Cell Rep. (2021) 34:108678. doi: 10.1016/j.celrep.2020.108678. PMID: 33503424 PMC8552368

[119] Oyama T Brashears CB Rathore R Benect-Hamilton H Caldwell KE Dirckx N . PHGDH inhibition and FOXO3 modulation drives PUMA-dependent apoptosis in osteosarcoma. Cell Death Dis. (2025) 16:89. doi: 10.1038/s41419-025-07378-6. PMID: 39934141 PMC11814296

[120] Wang D Wu L Cao Y Yang L Liu W E X . A novel mechanism of mTORC1-mediated serine/glycine metabolism in osteosarcoma development. Cell Signal. (2017) 29:107–14. doi: 10.1016/j.cellsig.2016.06.008. PMID: 27297361

[121] Li S Yu J Huber A Kryczek I Wang Z Jiang L . Metabolism drives macrophage heterogeneity in the tumor microenvironment. Cell Rep. (2022) 39:110609. doi: 10.1016/j.celrep.2022.110609. PMID: 35385733 PMC9052943

[122] Kishton RJ Sukumar M Restifo NP . Arginine arms T cells to thrive and survive. Cell Metab. (2016) 24:647–8. doi: 10.1016/j.cmet.2016.10.019. PMID: 27829132 PMC6327309

[123] Yang H Kim C Zou W . Metabolism and macrophages in the tumor microenvironment. Curr Opin Immunol. (2024) 91:102491. doi: 10.1016/j.coi.2024.102491. PMID: 39368171

[124] Liang P Li Z Chen Z Chen Z Jin T He F . Metabolic reprogramming of glycolysis, lipids, and amino acids in tumors: impact on CD8 + T cell function and targeted therapeutic strategies. FASEB J. (2025) 39:e70520. doi: 10.1096/fj.202403019R. PMID: 40249661

[125] Kurniawan H FranChina DG Guerra L Bonetti L - Baguet LS Grusdat M . Glutathione restricts serine metabolism to preserve regulatory T cell function. Cell Metab. (2020) 31:920–936.e7. doi: 10.1016/j.cmet.2020.03.004. PMID: 32213345 PMC7265172

[126] Loftus RM Assmann N Kedia-Mehta N O’Brien KL Garcia A Gillespie C . Amino acid-dependent cMyc expression is essential for NK cell metabolic and functional responses in mice. Nat Commun. (2018) 9:2341. doi: 10.1038/s41467-018-04719-2. PMID: 29904050 PMC6002377

[127] Lamas B Vergnaud-Gauduchon J Goncalves-Mendes N Perche O Rossary A Vasson MP . Altered functions of natural killer cells in response to L-arginine availability. Cell Immunol. (2012) 280:182–90. doi: 10.1016/j.cellimm.2012.11.018. PMID: 23399839

[128] Rodriguez PC Zea AH Culotta KS Zabaleta J Ochoa JB Ochoa AC . Regulation of T cell receptor CD3ζ chain expression byl-arginine. J Biol Chem. (2002) 277:21123–9. doi: 10.1074/jbc.M110675200. PMID: 11950832

[129] Patil MD Bhaumik J Babykutty S Banerjee UC Fukumura D . Arginine dependence of tumor cells: targeting a chink in cancer’s armor. Oncogene. (2016) 35:4957–72. doi: 10.1038/onc.2016.37. PMID: 27109103 PMC5457742

[130] Liao Y Chen J Yao H Zheng T Tu J Chen W . Single-cell profiling of SLC family transporters: uncovering the role of SLC7A1 in osteosarcoma. J Transl Med. (2025) 23:103. doi: 10.1186/s12967-025-06086-1. PMID: 39844299 PMC11752724

[131] Xiang D Han X Li J Zhang J Xiao H Li T . Combination of IDO inhibitors and platinum(IV) prodrugs reverses low immune responses to enhance cancer chemotherapy and immunotherapy for osteosarcoma. Mater Today Bio. (2023) 20:100675. doi: 10.1016/j.mtbio.2023.100675. PMID: 37304579 PMC10250924

[132] Husain Z Huang Y Seth P Sukhatme VP . Tumor-derived lactate modifies antitumor immune response: effect on myeloid-derived suppressor cells and NK cells. J Immunol. (2013) 191:1486–95. doi: 10.4049/jimmunol.1202702. PMID: 23817426

[133] Gao Y Wang Z Jin X Wang X Tao Y Huang S . Enhanced osteosarcoma immunotherapy via CaCO_3_ nanoparticles: remodeling tumor acidic and immune microenvironment for photodynamic therapy. Adv Healthc Mater. (2024) 13:2400538. doi: 10.1002/adhm.202400538. PMID: 38759954

[134] Zeng A Chen H Luo T Chen W Song Y Xu Y . Targeting OxLDL-mediated CD36 + CAF reprogramming potentiates PD-1 immunotherapy in osteosarcoma. Mol Cancer. (2025) 25:14. doi: 10.1186/s12943-025-02516-2. PMID: 41413558 PMC12829093

[135] Kano Y Soda K Nakamura T Saitoh M Kawakami M Konishi F . Increased blood spermine levels decrease the cytotoxic activity of lymphokine-activated killer cells: a novel mechanism of cancer evasion. Cancer Immunol Immunother. (2007) 56:771–81. doi: 10.1007/s00262-006-0229-4. PMID: 16972077 PMC11029869

[136] Janakiram NB Mohammed A Bryant T Zhang Y Brewer M Duff A . Potentiating NK cell activity by combination of Rosuvastatin and Difluoromethylornithine for effective chemopreventive efficacy against colon cancer. Sci Rep. (2016) 6:37046. doi: 10.1038/srep37046. PMID: 27841323 PMC5107958

[137] Qiu S Tan C Cheng D Yang Q . Identification and verification of a polyamine metabolism-related gene signature for predicting prognosis and immune infiltration in osteosarcoma. J Orthop Surg. (2025) 20:482. doi: 10.1186/s13018-025-05716-0. PMID: 40383808 PMC12087067

[138] Zhang C Yue C Herrmann A Song J Egelston C Wang T . STAT3 activation-induced fatty acid oxidation in CD8+ T effector cells is critical for obesity-promoted breast tumor growth. Cell Metab. (2020) 31:148–161.e5. doi: 10.1016/j.cmet.2019.10.013. PMID: 31761565 PMC6949402

[139] Lepple-Wienhues A Belka C Laun T Jekle A Walter B Wieland U . Stimulation of CD95 (Fas) blocks T lymphocyte calcium channels through sphingomyelinase and sphingolipids. Proc Natl Acad Sci. (1999) 96:13795–800. doi: 10.1073/pnas.96.24.13795. PMID: 10570152 PMC24144

[140] Zou Y Guo S Liao Y Chen W Chen Z Chen J . Ceramide metabolism-related prognostic signature and immunosuppressive function of ST3GAL1 in osteosarcoma. Transl Oncol. (2024) 40:101840. doi: 10.1016/j.tranon.2023.101840. PMID: 38029509 PMC10698579

[141] Ji R Wang Y Pan D Han J Wang Y Zheng S . NUCB2 inhibition antagonizes osteosarcoma progression and promotes anti-tumor immunity through inactivating NUCKS1/CXCL8 axis. Cancer Lett. (2024) 591:216893. doi: 10.1016/j.canlet.2024.216893. PMID: 38636892

[142] Wang Z Li B Li S Lin W Wang Z Wang S . Metabolic control of CD47 expression through LAT2-mediated amino acid uptake promotes tumor immune evasion. Nat Commun. (2022) 13:6308. doi: 10.1038/s41467-022-34064-4. PMID: 36274066 PMC9588779

[143] Yang D Chen Y He ZNT Wang Y Ke C Luo Y . Indoleamine 2,3-dioxygenase 1 promotes osteosarcoma progression by regulating tumor-derived exosomal miRNA hsa-miR-23a-3p. Front Pharmacol. (2023) 14:1194094. doi: 10.3389/fphar.2023.1194094. PMID: 37284323 PMC10239870

[144] Wu W Zhang Z Jing D Huang X Ren D Shao Z . SGLT2 inhibitor activates the STING/IRF3/IFN-β pathway and induces immune infiltration in osteosarcoma. Cell Death Dis. (2022) 13:523. doi: 10.1038/s41419-022-04980-w. PMID: 35662245 PMC9166744

[145] Uehara T Eikawa S Nishida M Kunisada Y Yoshida A Fujiwara T . Metformin induces CD11b+-cell-mediated growth inhibition of an osteosarcoma: implications for metabolic reprogramming of myeloid cells and anti-tumor effects. Int Immunol. (2019) 31:187–98. doi: 10.1093/intimm/dxy079. PMID: 30508092 PMC6440441

[146] Song Z Lu S Yang Y Chen Z Chen Y Cao J . Sonodynamic therapy augmented by glycolysis inhibition: a novel metabolic reprogramming strategy for enhanced osteosarcoma treatment. Natl Sci Rev. (2025) 12:nwaf365. doi: 10.1093/nsr/nwaf365. PMID: 41179741 PMC12573261

[147] He X Lin H Yuan L Li B . Combination therapy with L-arginine and α-PD-L1 antibody boosts immune response against osteosarcoma in immunocompetent mice. Cancer Biol Ther. (2017) 18:94–100. doi: 10.1080/15384047.2016.1276136. PMID: 28045576 PMC5362985

[148] Yuan X Yu S Zeng Z Yi L Yu B Zhu L . Hispidulin suppresses osteosarcoma by directly targeting FABP4 to disrupt lipid metabolism and inhibit the PI3K/AKT pathway. J Transl Med. (2025) 23:1062. doi: 10.1186/s12967-025-07128-4. PMID: 41057878 PMC12502341

[149] Zhou X Deng R Liao Z Huang X Huang J Yang H . Integrating metabolic modulation and nanomedicine for cancer immunotherapy. Adv Sci. (2025) 12:e10004. doi: 10.1002/advs.202510004. PMID: 40940612 PMC12561475

[150] Dai Y Wang J Liu Y Jiao G Gu Y Liu Y . Universal antibody‐engineered lipid nanoparticles potentiate chemo‐immunotherapy against triple‐negative breast cancer by reprogramming tumor cell metabolism. Adv Sci. (2026) 13:e18468. doi: 10.1002/advs.202518468. PMID: 41591256 PMC13045483

[151] Lacinski RA Dziadowicz SA Melemai VK Fitzpatrick B Pisquiy JJ Heim T . Spatial multiplexed immunofluorescence analysis reveals coordinated cellular networks associated with overall survival in metastatic osteosarcoma. Bone Res. (2024) 12:55. doi: 10.1038/s41413-024-00359-z. PMID: 39333065 PMC11436896

[152] Kelly CM Qin LX Whiting KA Richards AL Avutu V Chan JE . A phase II study of epacadostat and pembrolizumab in patients with advanced sarcoma. Clin Cancer Res. (2023) 29:2043–51. doi: 10.1158/1078-0432.CCR-22-3911. PMID: 36971773 PMC10752758

[153] O’Connell BC Hubbard C Zizlsperger N Fitzgerald D Kutok JL Varner J . Eganelisib combined with immune checkpoint inhibitor therapy and chemotherapy in frontline metastatic triple-negative breast cancer triggers macrophage reprogramming, immune activation and extracellular matrix reorganization in the tumor microenvironment. J Immunother Cancer. (2024) 12:e009160. doi: 10.1136/jitc-2024-009160. PMID: 39214650 PMC11367338

